# Discovery of 3-Amino-2-Hydroxypropoxyisoflavone Derivatives as Potential Anti-HCV Agents

**DOI:** 10.3390/molecules23112863

**Published:** 2018-11-02

**Authors:** Jin-Ching Lee, Chun-Kuang Lin, Chin-Kai Tseng, Yeh-Long Chen, Cherng-Chyi Tzeng, Chih-Hua Tseng

**Affiliations:** 1 Department of Biomedical Science and Environmental Biology, College of Life Science, Kaohsiung Medical University, Kaohsiung City 807, Taiwan; jclee@kmu.edu.tw (J.-C.L.); crystalsoul35@gmail.com (C.-K.L.); jimmytseng@biosidsco.com (C.-K.T.); 2 Department of Medicinal and Applied Chemistry, College of Life Science, Kaohsiung Medical University, Kaohsiung City 807, Taiwan; yeloch@kmu.edu.tw (Y.-L.C.); tzengch@kmu.edu.tw (C.-C.T.); 3 School of Pharmacy, College of Pharmacy, Kaohsiung Medical University, Kaohsiung City 807, Taiwan; 4 Department of Fragrance and Cosmetic Science, College of Pharmacy, Kaohsiung Medical University, Kaohsiung City 807, Taiwan; 5 Center for Infectious Disease and Cancer Research, Kaohsiung Medical University, Kaohsiung City 807, Taiwan; 6 Department of Pharmacy, Kaohsiung Municipal Ta-Tung Hospital, Kaohsiung City 807, Taiwan

**Keywords:** 3-amino-2-hydroxypropoxyisoflavones, ribavirin, hepatitis C virus, cytotoxicity

## Abstract

Synthesis and anti-hepatitis C virus (anti-HCV) effects of certain 3-amino-2-hydroxy-propoxy isoflavone derivatives, **6a**–**i**, were described. The known 3-(3,4-dimethoxyphenyl)-7-(oxiran-2-ylmethoxy)-4*H*-chromen-4-one (**5**) was reacted with substituted amines to give the desired isoflavone derivatives, **6a**–**i**. Among them, 7-{3-[(3,4-dimethoxy-phenethyl)amino]-2-hydroxypropoxy}-3-(3,4-dimethoxyphenyl)-4*H*-chromen-4-one (**6b**) was the most active, exhibiting approximately 2-fold higher anti-HCV effects than standard antiviral drug ribavirin (EC_50_ of 6.53 vs. 13.16 μM). In addition, compound **6b** was less cytotoxic than ribavirin. The selectivity index (SI) of **6b** is approximately 2.6-fold higher than ribavirin. The compounds **6e**, **6h**, and **6i** were also found to possess higher anti-HCV effects than ribavirin. Compound **6b** was found to inhibit the HCV RNA expression in Ava5 cells in a dose-dependent manner; furthermore, we found that the antiviral mechanism of compounds **6b**, **6e**, **6h**, and **6i** gave rise to induction of HO-1 expression. With the HO-1 promoter-based analysis, we found compounds **6b**, **6e**, **6h**, and **6i** induced HO-1 expression through increasing Nrf-2 binding activity. Taken together, compound **6b** may serve as a potential lead compound for developing novel anti-HCV agents.

## 1. Introduction

Hepatitis C virus (HCV) infection has significantly increased in the past decades and becomes a severe problem in liver diseases, including chronic hepatitis, cirrhosis, and hepatocellular carcinoma (HCC). Globally, an estimated 200 million people are infected with hepatitis C virus and more than 350,000 people die every year from HCV-related liver diseases [1,2,3]. In clinical therapies, there are still no approved vaccines for the treatment of HCV infection [4]. The therapeutic agents for HCV patients still present a drug-resistant problem, so the development of supplemental agents or more effective and safer agents is required for such therapy [5,6,7,8,9,10]. Recently, Andreev et al. [11] identified 1-benzyl-2-phenyl-4,5,6,7-tetrahydro-1*H*-indole (Compound **1**) as a potent anti-HCV agent which displayed EC_50_ values of 7.9 and 2.6 μM in genotype 1b and 2a, respectively. Kaushik-Basu et al. [12] reported that (3a*S*,8a*S*,*E*)-ethyl-4-(2-phenylhydrazono)-1-tosyldecahydro-cyclohepta[*b*]pyrrole-2-carboxylate (Compound **2**) exhibited EC_50_ values of 1.8 and 4.5 μM in genotype 1b and 2a, respectively. Zhong et al. [13] prepared certain quercetin analogues for anti-HCV evaluations and found 7-[(3-chlorobenzyl)oxy]-2-(3,4-dihydroxyphenyl)-3,5-dihydroxy-4*H*-chromen-4-one (Compound **3**) was the most potent, exhibiting an EC_50_ value of 3.8 μM. We have also synthesized certain naphtho [1,2-*d*]oxazole derivatives for anti-HCV evaluations and discovered 2-(furan-2-yl)-*N*-(4-methoxyphenyl)naphtho[1,2-*d*]oxazol-5-amine (Compound **4**) [14] to be the most active, exhibiting an EC_50_ value of 0.63 μM.

**Figure 1:**
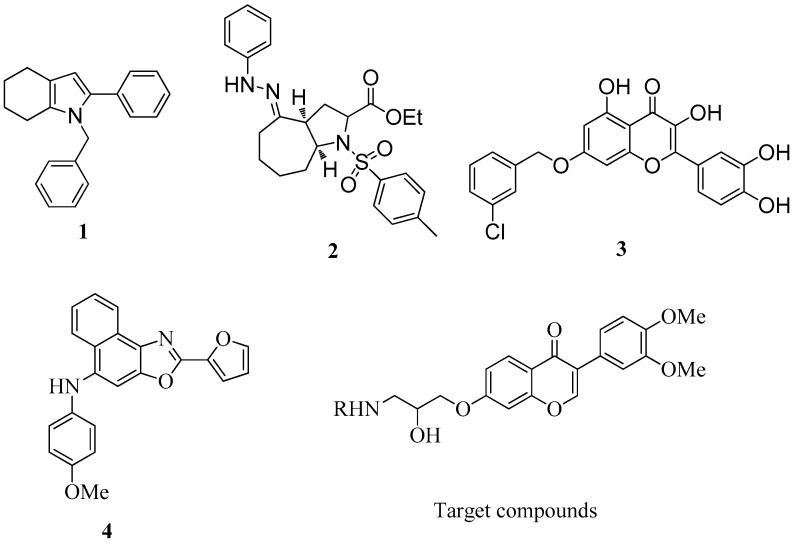
Structures of compounds **1**–**4**, and target compounds.

A number of natural isoflavonoids along with their synthetic analogues have been found to possess extensive biological activities including antiparasitic, anti-cancer, antiviral, anti-inflammatory, antioxidant, and anti-osteoporosis effects [15,16,17,18,19]. In order to further explore antiviral effects of isoflavonoids, we describe herein the synthesis of 3-amino-2-hydroxy-propoxyisoflavone derivatives (target compounds, Figure 1) and their evaluations related to the inhibition of HCV replication by inducing HO-1 expression.

## 2. Results and Discussion

### 2.1. Chemistry

#### Preparation of 3-Amino-2-Hydroxypropoxyisoflavone Derivatives

**Scheme 1:**
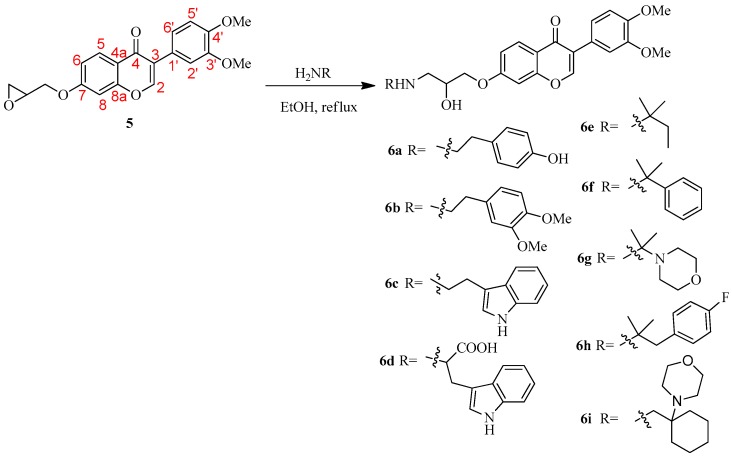
Synthesis of 3-amino-2-hydroxypropoxyisoflavone derivatives **6a**–**i**.

**Figure 2:**
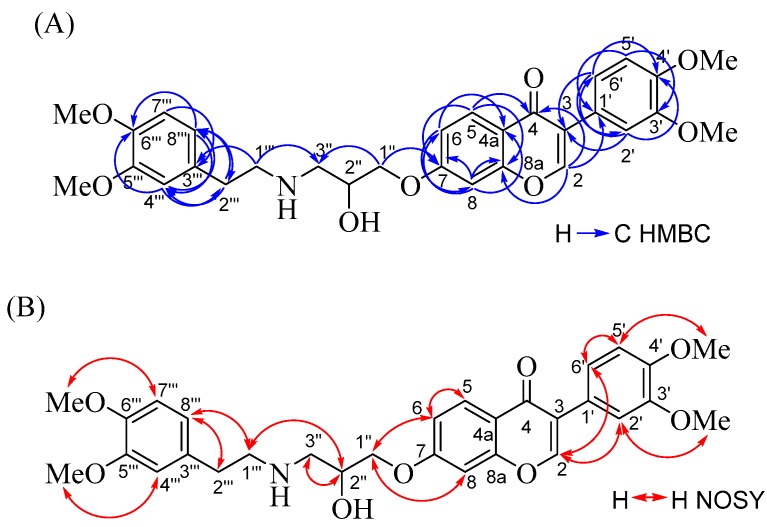
HMBC (**A**) and NOESY (**B**) correlations for 7-{3-[(3,4-dimethoxyphenethyl)amino]-2-hydroxypropoxy}-3-(3,4-dimethoxyphenyl)-*4H*-chromen-4-one (**6b**).

The desired 3-amino-2-hydroxypropoxyisoflavone derivatives, **6a**–**i**, have been prepared as described in Scheme 1*.* Reaction of 3-(3,4-dimethoxyphenyl)-7-(oxiran-2-ylmethoxy)-4*H*-chromen-4-one (**5**) [19] with 4-hydroxyphenylethylamine in ethanol gave 3-(3,4-dimethoxyphenyl)-7-{2-hydroxy-3-[(4-hydroxyphenethyl)amino]propoxy}-4*H*-chromen-4-one (**6a**) in 76% yield*.* 7-{3-[(3,4-Dimethoxyphenethyl)amino]-2-hydroxypropoxy}-3-(3,4-dimethoxyphenyl)-4*H*-chromen-4-one (**6b**) was obtained by the treatment of **5** with 3,4-dimethoxyphenylethylamine. The structure of **6b** was determined by ^13^C (100 MHz), ^1^H (400 MHz), heteronuclear multiple quantum coherence (HMQC), heteronuclear multiple bond correlation (HMBC), and nuclear Overhauser enhancement spectroscopy (NOESY) nuclear magnetic resonance (NMR) (Table 1). The spectra of **6b** (Table 1) revealed the presence of two sets of 3,4-dimethoxyphenyl-aromatic rings [δ_C_ 124.52 (C-1′) and 132.05 (C-3′″); δ_C_ 112.42 (C-2′)/δ_H_ 7.20 (2′-CH, d, *J* = 2.0 Hz) and δ_C_ 111.89 (C-4′″)/δ_H_ 6.74 (4′″-CH, m); δ_C_ 148.71 (C-3′) and 147.52 (C-5′″); δ_C_ 148.94 (C-4′) and 149.05 (C-6′″); δ_C_ 111.10 (C-5′)/δ_H_ 6.92 (5′-CH, d, *J* = 8.4 Hz) and δ_C_ 111.27 (C-7′″)/δ_H_ 6.80 (7′″-CH, d, *J* = 8.0 Hz); δ_C_ 120.98 (C-6′)/δ_H_ 7.04 (6′-CH, dd, *J* = 8.4, 2.0 Hz) and δ_C_ 20.55 (C-8′″)/δ_H_ 6.75 (m)], four methoxy groups [δ_C_ 55.89/δ_H_ 3.84 (s) for 3′-OMe, δ_C_ 55.91/δ_H_ 3.88 (s) for 4′-OMe, δ_C_ 55.82/δ_H_ 3.91 (s) for 5′″-OMe, and δ_C_ 55.87/δ_H_ 3.93 (s) for 6′″-OMe], *4H*-chromen-4-one moiety [δ_C_ 152.26 (C-2)/δ_H_ 7.94 (2-CH, s), δ_C_ 124.89 (C-3), δ_C_ 75.81 (C-4), δ_C_ 118.55 (C-4a), δ_C_ 127.75 (C-5)/δ_H_ 8.20 (5-CH, d, *J* = 8.8 Hz), δ_C_ 114.74 (C-6)/δ_H_ 6.99 (6-CH, d, *J* = 8.8, 2.4 Hz), δ_C_ 162.95 (C-7), δ_C_ 157.73 (C-8)/δ_H_ 6.86 (8-CH, d, *J* = 2.4 Hz), and δ_C_ 157.73 (C-8a)], and the 3-amino-2-hydroxypropoxy-spacer [δ_C_ 67.72 (C-1″)/δ_H_ 4.07 (1″-CH_2_, m), δ_C_ 70.95 (C-2″)/δ_H_ 4.07 (2″-CH, m), δ_C_ 51.32 (C-3″)/δ_H_ 2.91 (3″-CH_2_, m), δ_C_ 50.98 (C-1′″)/δ_H_ 2.91 and 2.78 (1′″-CH_2_, m), δ_C_ 35.85 (C-2′″)/δ_H_ 2.78 (2′″-CH_2_, m), and δ_H_ 2.23 (2″-OH and 3″-NH, br s)]. Its HMBC spectrum provided key correlations: (1) from H-2′ to C-3, 4′, 6′, and H-6′ to C-3, 2′, 4′ suggested the 3,4-dimethoxyphenyl group was attached to C-3 of the *4H*-chromen-4-one moiety; (2) from H-1″ to C-7, 3″, H-1′″ to C-3″, 3′″ indicated the other 3,4-dimethoxyphenyl group was attached to C-2′″ of the 3-amino-2-hydroxypropoxy-spacer and the spacer was attached to C-7 of the *4H*-chromen-4-one moiety (Figure 2A). The relative connection was established according to nuclear Overhauser effect (NOE) correlations between H-2/H-2′, 6′; H-1″/H-6, 8; H-1′″/H-2″, 8′″; and H-2′″/H-8′″ in the NOESY experiment (Figure 2B). Accordingly, compounds **6c**–**6i** have been prepared by amination of **5** in a yield of 65–83%. The structure of **6a**–**i** was determined by NMR (^1^H and ^13^C) (spectra data can be found in Supplementary Materials) and further confirmed by elemental analysis.

### 2.2. Biological Activities

#### 2.2.1. Anti-HCV Activities and Cytotoxicities

The anti-HCV and cytotoxicities of 3-amino-2-hydroxypropoxyisoflavone derivatives are summarized in Table 2. Ava-5 cells were treated with compounds **6a**–**i** or the positive ribavirin for three days, and then analyzed through firefly luciferase assay. The concentration that inhibited 50% HCV replication (EC_50_), the concentration that inhibited 50% cell growth (CC_50_), and the selectivity index (SI: CC_50_/EC_50_) of compounds were determined with ribavirin as a positive control. Results indicated that compounds **6b**, **6e**, **6h** and **6i** were more active than ribavirin. Among them, compound **6b** was the most active, exhibiting approximately 2-fold more anti-HCV activity (EC_50_ of 6.53 μM) than that of ribavirin (EC_50_ = 13.16 μM). In addition, compound **6b** was less cytotoxic than ribavirin. The selectivity index (SI) of **6b** is approximately 2.6-fold higher than that of ribavirin (21.08 vs. 8.08).

#### 2.2.2. Compound **6b** Reduced HCV Replication in HCV-Infected Ava-5 Cells

**Figure 3:**
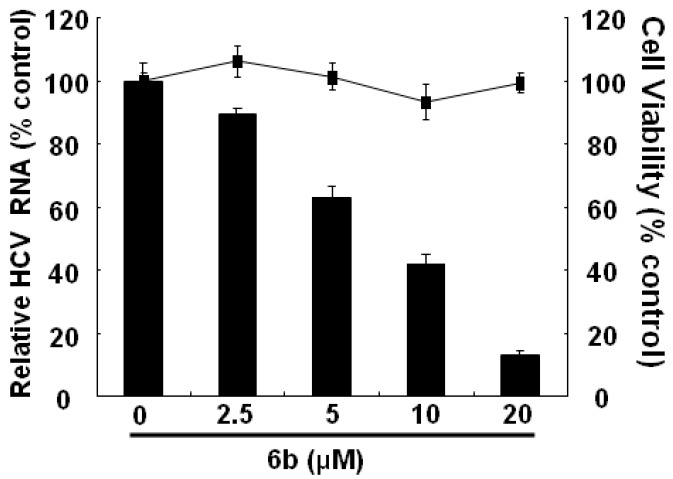
Inhibition of HCV RNA expression in HCV-infected Ava-5 cells by **6b**. Ava-5 cells were treated with 2.5, 5, 10 and 20 μM of **6b** for 3 days. Total RNA was extracted and quantified HCV RNA levels by RT-qPCR. HCV RNA expression was normalized by cellular GAPDH mRNA. Treatment with 0.1% DMSO served as a mock control. The results are expressed as the means ± standard deviations (SD) of triplicate experiments.

**Figure 4:**
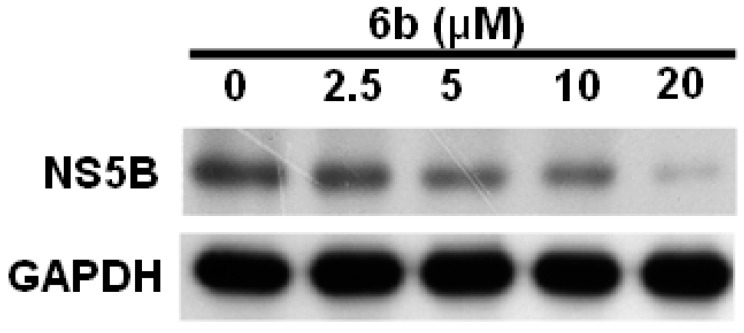
Inhibition of HCV protein synthesis in Ava-5 cells by **6b**. Ava-5 cells were treated with 2.5, 5, 10 and 20 μM of **6b** for 3 days. Total cell lysate was collected for performing western blotting to analyze HCV protein synthesis. Levels of GAPDH were used as equal loading control.

To further confirm the anti-HCV effect of compound **6b**, we treated compound **6b** at indicated concentrations in Ava-5 cells for 3 days. Both western blotting and RT-qPCR were performed to determine the resultant activity of compound **6b** against HCV replication showing that compound **6b** dose-dependently reduced HCV protein synthesis and RNA replication without cell cytotoxicity in Ava5 cells. Treatment of 0.1% dimethyl sulfoxide (DMSO) served as a mock control on inhibition of HCV replication (Figure 3 and Figure 4).

#### 2.2.3. Isoflavones Reduced HCV Replication through Inducing HO-1 Protein Expression

**Figure 5:**
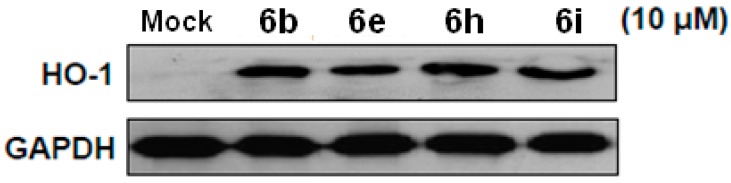
Compounds induced HO-1 protein expression in Ava-5 cells. Compound **6b**, **6e**, **6h** and **6i** induced HO-1 protein expression. Ava-5 cells were treated with compounds at 10 μM for 3 days. The cell lysate was subjected to western blotting with anti-HO-1 and anti-GAPDH antibodies.

In our previous studies, we found that induction of HO-1 protein level could suppress HCV replication [14,20]. To determine whether compounds **6b**, **6e**, **6h** and **6i** have impact on HO-1 protein expression in Ava-5 cells, we treated these compounds at 10 μM in Ava5 cells. Results indicated that compounds **6b**, **6e**, **6h** and **6i** could induce HO-1 protein level in Ava-5 cells, compared with the DMSO-treated Ava5 cells (Figure 5).

#### 2.2.4. Isoflavones Up-Regulates Nrf2 Transactivating HO-1 Expression to Inhibit HCV Replication

**Figure 6:**
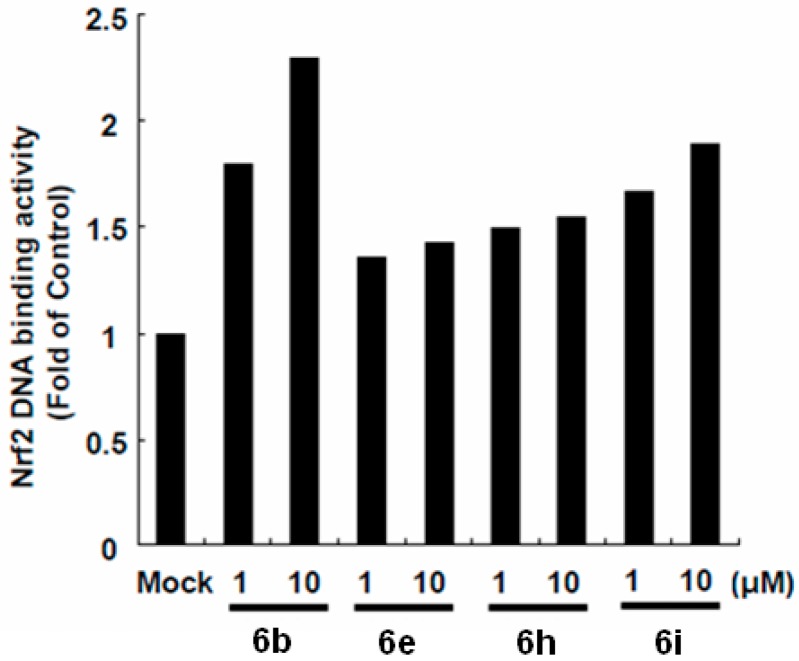
Isoflavones inhibited HCV replication by upregulating Nrf2 expression. Compound **6b**, **6e**, **6h** and **6i** stimulated ARE transactivation in Ava-5 cells. The antioxidant response reporter plasmid, p3xARE-Luc, was transfected into Ava-5 cells and then treated with isoflavones (1 and 10 μM) for 3 days. The relative induction of antioxidant activity was determined by luciferase assay. The activity of untreated Ava-5 cells was considered to be 1.

Heme oxygenase 1 expression is regulated by the transcription factors Nrf2, Keap1, and Bach1 through the binding of ARE in its promoter region [21,22,23]; therefore, we determined whether isoflavones-mediated HO-1 induction was dependent on ARE transactivation by treating p3xARE-Luc-transfected ava-5 cells with increasing concentrations of sulforaphane (SFN) for 3 days. As shown in Figure 6, compounds **6b**, **6e**, **6h** and **6i** increased ARE-mediated luciferase activity. Taken together, these results suggest that the anti-HCV activity of isoflavones were dependent on Nrf2-mediated HO-1 induction.

## 3. Experimental

### 3.1. Materials and Methods

#### 3.1.1. Chemical Reactions

##### General

Melting points were determined on an Electrothermal IA9100 melting point apparatus and are uncorrected. Nuclear magnetic resonance (^1^H) spectra were recorded on a Varian-Unity-400 spectrometer (Varian, Palo Alto, CA, USA). Chemical shifts were expressed in parts per million (δ) with tetramethylsilane (TMS) as an internal standard. Thin-layer chromatography was performed on silica gel 60 F-254 plates purchased from E. Merck and Co. (Darmstadt, Germany). The elemental analyses were performed in the Instrument Center of Ministry of Science and Technology at National Cheng-Kung University and National Taiwan University using Heraeus CHN-O Rapid EA (Heraeus, Waltham, MA, USA), and all values are within ±0.4% of the theoretical compositions.

##### General Procedure for the Preparation of 3-Amino-2-Hydroxypropoxyisoflavone Compounds **6a**–**i**

To a suspension of **5** (1.0 mmol) in ethanol (15 mL) was added substituted amines (3.0 mmol). The reaction mixture was refluxed for 3 h (TLC monitoring). The solvent was removed in vacuo and the residue suspended in H_2_O (20 mL). The crude product was purified by flash chromatography on silica gel and recrystallized from MeOH to afford the 3-amino-2-hydroxypropoxyisoflavone products.

*3-(3,4-Dimethoxyphenyl)-7-{2-hydroxy-3-[(4-hydroxyphenethyl)amino]propoxy}-4H-chromen-4-one* (**6a**): Yield 76%. Mp: 117–118 °C. ^1^H NMR (400 MHz, DMSO-*d*_6_): δ 9.18 (br s, 1H), 8.46 (s, 1H, H-2), 8.04 (d, *J* = 8.8 Hz, 1H, H-5), 7.21 (d, *J* = 2.0 Hz, 1H, H-2′), 7.17–7.14 (m, 2H, H-8, H-6′), 7.08 (dd, 1H, *J* = 8.8, 2.4 Hz, H-6), 7.02 (d, *J* = 8.4 Hz, 1H, H-5′), 7.01–6.98 (m, 2H), 6.68–6.65 (m, 2H), 5.17 (br s, 1H), 4.15–4.11 (m, 1H), 4.05–4.01 (m, 1H), 3.96–3.92 (m, 1H), 3.79 (s, 6H), 2.75–2.59 (m, 6H). ^13^C NMR (100 MHz, DMSO-*d*_6_): δ 174.66 (C-4), 163.16 (C-7), 157.38 (C-8a), 155.48, 153.71 (C-2), 148.66 (C-4′), 148.30 (C-3′), 130.24, 129.46 (2C), 126.98 (C-5), 124.39 (C-1′), 123.48 (C-3), 121.27 (C-6′), 117.58 (C-4a), 115.17 (C-6), 115.06 (2C), 112.74 (C-2′), 111.54 (C-5′), 101.09 (C-8), 71.60, 67.84, 55.54 (3′-OMe-3′, 4′-OMe), 51.99, 51.44, 34.91. Anal. calcd. for C_28_H_29_NO_7_∙1.2H_2_O: C 65.54, H 6.17, N 2.73; found: C 65.50, H 6.06, N 2.59.

*7-{3-[(3,4-Dimethoxyphenethyl)amino]-2-hydroxypropoxy}-3-(3,4-dimethoxyphenyl)-4H-chromen-4-one* (**6b**): Yield 69%. Mp: 134–135 °C. ^1^H NMR (400 MHz, CDCl_3_): δ 8.20 (d, 1H, *J* = 8.8 Hz, H-5), 7.94 (s, 1H, H-2), 7.20 (d, 1H, *J* = 2.0 Hz, H-2′), 7.04 (dd, 1H, *J* = 8.4, 2.0 Hz, H-6′), 6.99 (dd, 1H, *J* = 8.8, 2.4 Hz, H-6), 6.92 (d, 1H, *J* = 8.4 Hz, H-5′), 6.86 (d, 1H, *J* = 2.4 Hz, H-8), 6.80 (d, 1H, *J* = 8.0 Hz), 6.77–6.74 (m, 2H), 4.10–4.04 (m, 3H), 3.93 (s, 3H), 3.91 (s, 3H), 3.88 (s, 3H), 3.86 (s, 3H), 2.99–2.74 (m, 6H), 2.23 (br s, 2H, OH and NH). ^13^C NMR (100 MHz, CDCl_3_) δ 175.81 (C-4), 162.95 (C-7), 157.73 (C-8a), 152.26 (C-2), 149.10, 148.99 (C-4′), 148.77 (C-3′), 147.57, 132.14, 127.81 (C-5), 124.94 (C-1′), 124.57 (C-3), 121.02 (C-6′), 120.59, 118.62 (C-4a), 114.77 (C-6), 112.48 (C-2′), 111.95, 111.34 (C-5′), 111.16, 100.89 (C-8), 70.98, 67.78, 55.95, 55.93, 55.91 (4′-OMe), 55.86 (3′-OMe), 51.31, 51.02, 35.95. Anal. calcd. for C_30_H_33_NO_8_: C 67.28, H 6.21, N 2.62; found: C 66.91, H 6.31, N 2.57.

*7-{3-{[2-(1H-Indol-3-yl)ethyl]amino}-2-hydroxypropoxy}-3-(3,4-dimethoxyphenyl)-4H-chromen-4-one* (**6c**): Yield 65%. Mp: 178–179 °C. ^1^H NMR (400 MHz, DMSO-*d*_6_): δ 10.80 (br s, 1H), 8.45 (s, 1H, H-2), 8.04 (d, 1H, *J* = 8.8 Hz, H-5), 7.53–7.51 (m, 1H), 7.34–7.32 (m, 1H), 7.21 (d, 1H, *J* = 2.0 Hz, H-2′), 7.17–7.14 (m, 3H), 7.09–7.04 (m, 2H), 7.01 (d, 1H, *J* = 8.4 Hz, H-5′), 6.98–6.94 (m, 1H), 5.14 (br s, 1H), 4.17–4.13 (m, 1H), 4.06–4.02 (m, 1H), 3.98–3.93 (m, 1H), 3.79 (s, 6H), 2.86 (br s, 4H), 2.77–2.66 (m, 2H). ^13^C NMR (100 MHz, DMSO-*d*_6_): δ 174.66 (C-4), 163.19 (C-7), 157.39 (C-8a), 153.70 (C-2), 148.65 (C-4′), 148.30 (C-3′), 136.26, 127.28, 126.97 (C-5), 124.39 (C-1′), 123.47 (C-3), 122.62, 121.27 (C-6′), 120.87, 118.34, 118.16, 117.57 (C-4a), 115.17 (C-6), 112.72 (C-2′), 112.48, 111.54 (C-5′), 111.37, 101.09 (C-8), 71.66, 67.96, 55.56 (4′-OMe), 55.54 (3′-OMe), 52.11, 50.27, 25.51. Anal. calcd. for C_30_H_30_N_2_O_6_∙0.2H_2_O: C 69.53, H 5.92, N 5.41; found: C 69.35, H 5.86, N 5.33.

*3-{{3-{[3-(3,4-Dimethoxyphenyl)-4-oxo-4H-chromen-7-yl]oxy}-2-hydroxypropyl}amino}-2-(1H-indol-3-yl)propanoic acid* (**6d**): Yield 71%. Mp: 221–222 °C. ^1^H NMR (400 MHz, DMSO-*d*_6_): δ 10.91 (br s, 1H), 8.44 (s, 1H, H-2), 8.02 (d, 1H, *J* = 8.8 Hz, H-5), 7.57 (d, 1H, *J* = 7.6 Hz, H-2′), 7.33 (d, 1H, *J* = 7.6 Hz), 7.22–6.94 (m, 8H), 4.07–3.96 (3m, 3H), 3.78 (s, 6H), 3.24–2.74 (m, 6H). ^13^C NMR (100 MHz, DMSO-*d*_6_): δ 174.68 (C-4), 172.50, 162.90 (C-7), 157.34 (C-8a), 153.77 (C-2), 148.66 (C-4′), 148.30 (C-3′), 136.21, 127.35 (C-5), 127.00, 124.37 (C-1′), 123.91, 123.50 (C-3), 121.29 (C-6′), 121.00, 118.55, 118.39, 117.69 (C-4a), 115.13 (C-6), 112.69 (C-2′), 111.52 (C-5′), 111.40, 109.69, 101.14 (C-8), 70.96, 66.42, 62.27, 55.57 (4′-OMe), 55.55 (3′-OMe), 49.66, 27.15. Anal. calcd. for C_31_H_30_N_2_O_8_∙1.5H_2_O: C 63.57, H 5.69, N 4.78; found: C 63.22, H 5.54, N 4.81.

*3-(3,4-Dimethoxyphenyl)-7-(2-hydroxy-3-(tert-pentylamino)propoxy)-4H-chromen-4-one* (**6e**): Yield 80%. Mp: 111–112 °C. ^1^H NMR (400 MHz, CDCl_3_): δ 8.21 (d, 1H, *J* = 9.2 Hz, H-5), 7.95 (s, 1H, H-2), 7.21 (d, 1H, *J* = 2.0 Hz, H-2′), 7.06–7.01 (m, 2H, H-6, H-6′), 6.92 (d, 1H, *J* = 8.4 Hz, H-5′), 6.88 (d, 1H, *J* = 2.4 Hz, H-8), 4.13–4.05 (m, 3H), 3.93 (s, 3H), 3.91 (s, 3H), 2.91 (dd, 1H, *J* = 12.0, 3.2 Hz), 2.70 (dd, 1H, *J* = 12.0, 7.6 Hz), 2.56 (br s, 1H), 1.48 (q, 2H, *J* = 8.0 Hz), 1.11 (s, 6H), 0.90 (t, 3H, *J* = 8.0 Hz). ^13^C NMR (100 MHz, CDCl_3_): δ 175.85 (C-4), 163.04 (C-7), 157.78 (C-8a), 152.27 (C-2), 149.08 (C-4′), 148.75 (C-3′), 127.89 (C-5), 124.92 (C-1′), 124.58 (C-3), 121.01 (C-6′), 118.59 (C-4a), 114.83 (C-6), 112.48 (C-2′), 111.13 (C-5′), 100.87 (C-8), 71.00, 67.98, 55.94 (4′-OMe), 55.92 (3′-OMe), 53.49, 44.05, 33.20, 26.23, 26.18, 8.24. Anal. calcd. for C_26_H_33_NO_6_∙0.5H_2_O: C 68.21, H 7.39, N 3.02; found: C 68.55, H 7.30, N 3.07.

*3-(3,4-Dimethoxyphenyl)-7-{2-hydroxy-3-[(2-phenylpropan-2-yl)amino]propoxy}-4H-chromen-4-one* (**6f**): Yield 68%. Mp.: 131–132 °C. ^1^H NMR (400 MHz, CDCl_3_): δ 8.18 (d, 1H, *J* = 8.8 Hz, H-5), 7.94 (s, 1H, H-2), 7.46–7.43 (m, 2H), 7.36–7.32 (m, 2H), 7.25–7.21 (m, 1H), 7.19 (d, 1H, *J* = 2.0 Hz, H-2′), 7.04 (dd, 1H, *J* = 8.0, 2.0 Hz, H-6′), 6.96 (dd, 1H, *J* = 9.2, 2.4 Hz, H-6), 6.92 (d, 1H, *J* = 8.4 Hz, H-5′), 6.83 (d, 1H, *J* = 2.4 Hz, H-8), 4.03–3.96 (m, 3H), 3.92 (s, 3H), 3.91 (s, 3H), 2.65 (dd, 1H, *J* = 12.0, 3.6 Hz), 2.48 (dd, 1H, *J* = 12.0, 7.2 Hz), 2.07 (br s, 1H, NH), 1.51 (s, 6H). ^13^C NMR (100 MHz, CDCl_3_): δ 175.85 (C-4), 162.98 (C-7), 157.73 (C-8a), 152.25 (C-2), 149.01 (C-4′), 148.68 (C-3′), 146.93, 128.30 (2C), 127.72 (C-5), 126.53, 125.73 (2C), 124.88 (C-1′), 124.53 (C-3), 120.97 (C-6′), 118.50 (C-4a), 114.80 (C-6), 112.38 (C-2′), 111.05 (C-5′), 100.76 (C-8), 70.98, 68.73, 55.90 (3′-OMe, 4′-OMe), 55.76, 45.14, 29.68, 29.37. Anal. calcd. for C_29_H_31_NO_6_: C 71.15, H 6.38, N 2.86; found: C 71.01, H 6.34, N 2.57.

*3-(3,4-Dimethoxyphenyl)-7-{2-hydroxy-3-[(2-morpholinopropan-2-yl)amino]propoxy}-4H-chromen-4-one* (**6g**): Yield 78%. Mp: 108–109 °C. ^1^H NMR (400 MHz, CDCl_3_): δ 8.19 (d, 1H, *J* = 8.8 Hz, H-5), 7.93 (s, 1H, H-2), 7.19 (d, 1H, *J* = 2.0 Hz, H-2′), 7.04-6.98 (m, 2H, H-6, H-6′), 6.91 (d, 1H, *J* = 8.4 Hz, H-5′), 6.85 (d, 1H, *J* = 2.4 Hz, H-8), 4.32–4.30 (m, 1H), 4.15–4.07 (m, 2H), 3.92 (s, 3H), 3.91 (s, 3H), 3.78–3.72 (m, 4H), 3.15–3.11 (m, 1H), 2.91–2.83 (m, 2H), 2.67–2.52 (m, 5H) 1.17 (s, 3H), 1.10 (s, 3H). ^13^C NMR (100 MHz, CDCl_3_): δ 175.78 (C-4), 162.79 (C-7), 157.72 (C-8a), 152.29 (C-2), 149.07 (C-4′), 148.72 (C-3′), 127.80 (C-5), 124.90 (C-1′), 124.49 (C-3), 121.00 (C-6′), 118.64 (C-4a), 114.72 (C-6), 112.43 (C-2′), 111.10 (C-5′), 100.86 (C-8), 70.56, 67.33 (2C), 66.54, 56.49, 56.14, 55.93 (4′-OMe), 55.90 (3′-OMe), 52.19, 45.82 (2C), 22.37, 20.81. Anal. calcd. for C_28_H_36_N_2_O_7_∙0.5H_2_O: C 64.46, H 7.16, N 5.37; found: C 64.26, H 7.18, N 5.20.

*3-(3,4-Dimethoxyphenyl)-7-{3-{[1-(4-fluorophenyl)-2-methylpropan-2-yl]amino}-2-hydroxypropoxy}-4H-chromen-4-one* (**6h**): Yield 83%. Mp: 151–152 °C. ^1^H NMR (400 MHz, DMSO-*d*_6_): δ 8.46 (s, 1H, H-2), 8.04 (d, 1H, *J* = 8.8 Hz, H-5), 7.23–7.14 (m, 5H), 7.09 (dd, 1H, *J* = 8.8, 2.4 Hz, H-6), 7.07–7.01 (m, 3H), 5.10 (br s, 1H), 4.17 (dd, 1H, *J* = 10.0, 4.0 Hz), 4.06 (dd, 1H, *J* = 10.0, 6.0 Hz), 3.89–3.86 (m, 1H), 3.79 (s, 6H), 2.77–2.65 (m, 2H), 2.62 (s, 2H), 0.96 (s, 3H), 0.95 (s, 3H). ^13^C NMR (100 MHz, DMSO-*d*_6_): δ 174.63 (C-4), 163.20 (C-7), 160.77 (*J* = 240.3 Hz), 157.36 (C-8a), 153.68 (C-2), 148.48 (C-4′), 148.31 (C-3′), 134.84 (*J* = 3.1 Hz), 132.06 (2C, *J* = 7.6 Hz), 126.96 (C-5), 124.38 (C-1′), 123.47 (C-3), 121.27 (C-6′), 117.55 (C-4a), 115.15 (C-6), 114.26 (2C, *J* = 20.5 Hz), 112.76 (C-2′), 111.57 (C-5′), 101.08 (C-8), 71.52, 68.87, 55.56 (4′-OMe), 55.55 (3′-OMe), 52.49, 45.37, 44.61, 26.66, 26.58. Anal. calcd. for C_30_H_32_FNO_6_∙0.2H_2_O: C 68.61, H 6.22, N 2.67; found: C 68.49, H 6.21, N 2.26.

*3-(3,4-Dimethoxyphenyl)-7-{2-hydroxy-3-{[(1-morpholinocyclohexyl)methyl]amino}propoxy}-4H-chromen-4-one* (**6i**): Yield 74%. Mp: 67–68 °C. ^1^H NMR (400 MHz, CDCl_3_): δ 8.19 (d, 1H, *J* = 8.8 Hz, H-5), 7.93 (s, 1H, H-2), 7.19 (d, 1H, *J* = 2.0 Hz, H-2′), 7.04–6.99 (m, 2H, H-6, H-6′), 6.91 (d, 1H, *J* = 8.0 Hz, H-5′), 6.86 (d, 1H, *J* = 2.4 Hz, H-8), 4.34–4.32 (m, 1H), 4.15–4.07 (m, 2H), 3.92 (s, 3H), 3.91 (s, 3H), 3.76–3.69 (m, 4H), 3.14–3.05 (m, 2H), 2.84 (dd, 1H, *J* = 12.0, 8.8 Hz), 2.69–2.57 (m, 5H), 1.69–1.19 (m, 12H). ^13^C NMR (100 MHz, CDCl_3_): δ 175.78 (C-4), 162.80 (C-7), 157.72 (C-8a), 152.28 (C-2), 149.07 (C-4′), 148.73 (C-3′), 127.81 (C-5), 124.90 (C-1′), 124.49 (C-3), 121.00 (C-6′), 118.64 (C-4a) 114.72 (C-6), 112.44 (C-2′), 111.11 (C-5′), 100.87 (C-8), 70.60, 67.75 (2C), 66.52, 58.22, 55.92 (4′-OMe), 55.90 (3′-OMe), 52.33, 50.01, 45.41 (2C), 29.99, 29.63, 25.77, 22.07, 21.98. Anal. calcd. for C_31_H_40_N_2_O_7_∙2.6H_2_O: C 62.10, H 7.60, N 4.67; found: C 61.88, H 7.20, N 4.47.

#### 3.1.2. Cytotoxicity and Antiviral Activity Assays

##### Compounds

Compounds were dissolved in DMSO at 10 mM and then diluted in culture medium.

##### Cell

Ava5 cells, an engineered HCV subgenomic replicon cell line, were cultured in Dulbecco′s modified Eagle′s medium (DMEM) with 10% heat-inactivated fetal bovine serum, 1% antibiotic–antimycotic, and 1% non-essential amino acids. Ava5 cells were maintained in DMEM with 1 mg mL^−1^ G418 to maintain the stable expression of replicon.

##### Cytotoxicity Assays

For cytotoxicity tests, run in parallel with antiviral assays, plates at an initial density of (5 × 10^3^ cells/well) were treated with or without serial dilutions of test compounds. Cell viability was determined after 72 h at 37 °C in a humidified CO_2_ (5%) atmosphere by the (2,3-bis[2-methyloxy-4-nitro-5-sulfophenyl]-2*H*-tetrazolium-5-carboxanilide) (XTT) method [24].

##### Transfection and Luciferase Activity Assay

Ava5 cells were transfected with the HO-1 promoter-driven luciferase plasmid, pHO-1-Luc, using the T-pro^TM^ transfection reagent (Ji-Feng Biotechnology Co., Ltd., Taipei, Taiwan) according to the manufacturer′s instructions. The transfected cells were treated with compounds at various concentrations for 3 days. Each transfection complex contains 0.1 μg pSEAP, a secreted alkaline phosphatase (SEAP) expression vector, for normalization luciferase activity serving as a transfection control. The luciferase activity assay was performed using the Bright-Glo Luciferase assay system (Promega) (Madison, WI, USA) according to the manufacturer′s instructions.

##### Immunoblot Analysis

Ava5 cells were seeded in 24-well plates at a density of 5 × 10^4^ cells per well overnight and treated with indicated reagent at proper concentrations for 3 days. Cells were washed with cold phosphate buffer saline (PBS) and lysed by radioimmunoprecipitation assay (RIPA) lysis buffer (50 mM Tris-HCl, pH 7.5, 150 mM NaCl, 1% Nonidet P-40, 2 mM EDTA, 1 mM EGTA, 1 mM NaVO_3_, 10 mM NaF, 1 mM DTT, 1 mM PMSF, 25 μg/mL aprotinin, and 25 μg/mL leupeptin) and stored at −20 °C. The protein concentration was determined by the Bradford method. Ten μg protein were separated by 10% SDS-PAGE and transferred onto a polyvinylidene difluorid (PVDF) membrane. The membrane was blocked with 5% non-fat dried milk and incubated with specific antibodies against NS5B (1:5000; Abcam Cambridge, MA, USA), glyceraldehyde-3-phosphate dehydrogenase (GAPDH) and anti-HO-1 (1:3000, Abcam Cambridge, MA, USA). Antibodies were diluted in 5% milk containing Tris-buffered saline (TBS) and 0.5% Tween. The blotting signal was developed using an ECL detection kit (PerkinElmer, Norwalk, CT, USA) and was counted by the software Quantity One (Bio-Rad, Foster, CA, USA).

## 4. Conclusions

We have synthesized and evaluated 3-amino-2-hydroxypropoxyisoflavone derivatives for their inhibitory activities of anti-HCV replication. These compounds exhibited better EC_50_ and SI values than ribavirin upon the antiviral experiment. Among them, 7-{3-[(3,4-dimethoxyphenethyl)amino]-2-hydroxypropoxy}-3-(3,4-dimethoxyphenyl)-4*H*-chromen-4-one (**6b**) exhibited the most potent activity against HCV replication. By the determination of antiviral mechanism, the results indicated that compounds **6b**, **6e**, **6h**, and **6i** reduced HCV replication through Nrf2-mediated HO-1 induction. Further studies on the structural optimization are ongoing.

## Figures and Tables

**Table 1:** ^13^C (100 MHz), ^1^H (400 MHz), *HC* HMBC and NOESY nuclear magnetic resonance (NMR) Data for 7-{3-[(3,4-dimethoxy phenethyl)amino]-2-hydroxypropoxy}-3-(3,4-dimethoxyphenyl)-*4H*-chromen-4-one (**6b**) in CDCl_3_. ^a^

^13^C		^1^H ^b^		
Position	δ_C_ (mult.)	δ_H_ (mult., *J*, Hz)	*HC* HMBC	NOESY
2	152.26 (CH)	7.94 (s)	4, 8a, 1′	2′, 6′
3	124.89 (C)			
4	175.81 (C)			
4a	118.55 (C)			
5	127.75 (CH)	8.20 (d, 8.8)	4, 7, 8a	6
6	114.74 (CH)	6.99 (dd, 8.8, 2.4)	4a, 8	5, 1″
7	162.95 (C)			
8	100.84 (CH)	6.86 (d, 2.4)	4a, 6, 7, 8a	1″
8a	157.73 (C)			
1′	124.52 (C)			
2′	112.42 (CH)	7.20 (d, 2.0)	3, 4′, 6′	2, 3′-OMe
3′	148.71 (C)			
3′-OMe	55.89 (CH_3_)	3.84 (s)		2′
4′	148.94 (C)			
4′-OMe	55.91 (CH_3_)	3.88 (s)		5′
5′	111.10 (CH)	6.92 (d, 8.4)	1′, 3′	6′, 4′-OMe
6′	120.98 (CH)	7.04 (dd, 8.4, 2.0)	3, 2′, 4′	2, 5′
1″	67.72 (CH_2_)	4.07 (m), overlapped	7, 3″	6, 8
2″	70.95 (CH)	4.07 (m), overlapped		3″, 1′″
2″-OH		2.23 (br s), overlapped		
3″	51.32 (CH_2_)	2.91 (m), overlapped		2″
3″-NH		2.23 (br s), overlapped		
1′″	50.98 (CH_2_)	2.91 and 2.78 (m), overlapped	3″, 3′″	2″, 8′″
2′″	35.85 (CH_2_)	2.78 (m), overlapped	4′″, 8′″	8′″
3′″	132.05 (C)			
4′″	111.89 (CH)	6.74 (m), overlapped	2′″, 6′″, 8′″	5′″-OMe
5′″	147.52 (C)			
5′″-OMe	55.82 (CH_3_)	3.91 (s)		4′″
6′″	149.05 (C)			
6′″-OMe	55.87 (CH_3_)	3.93 (s)		7′″
7′″	111.27 (CH)	6.80 (d, 8.0)	3′″	6′″-OMe
8′″	120.55 (CH)	6.75 (m), overlapped	2′″, 4′″, 6′″	1′″, 2′″

^a^ s: Singlet, d: Doublet, dd: Doublet of doublets, br s: Broad singlet, m: Multiple, ov: Overlapped; ^b^ The ^13^C and ^1^H correlations were confirmed by the heteronuclear multiple quantum coherence (HMQC) experiment.

**Table 2:** Antiviral activities and cytotoxicities of isoflavone derivatives.

Compound	EC_50_ (μM) ^a^	CC_50_ (μM) ^b^	SI ^c^
**6a**	>20	71.65 ± 4.44	<3.58
**6b**	6.53 ± 0.57	137.68 ± 6.91	21.08
**6c**	16.32 ± 0.95	36.93 ± 0.46	2.26
**6d**	>20	98.84 ± 3.67	<4.94
**6e**	8.14 ± 1.74	87.91 ± 2.13	10.80
**6f**	14.31 ± 0.84	47.19 ± 2.74	3.30
**6g**	>20	143.57 ± 3.82	<7.17
**6h**	9.35 ± 0.97	110.98 ± 4.39	11.87
**6i**	10.71 ± 0.87	155.87 ± 1.58	14.55
**ribavirin**	13.16 ± 1.63	106.27 ± 3.69	8.08

^a^ The EC_50_ is the concentration of the compound resulting in a 50% inhibition in virus production; ^b^ The CC_50_ is the concentration of the compound causing a 50% growth inhibition of uninfected ava-5 cells; ^c^ SI: Selectivity index. SI = CC_50_/IC_50_.

## Supplementary Materials

The supplementary materials are available online.
