# Supplementary material for: Discovery of 3-Amino-2-Hydroxypropoxyisoflavone Derivatives as Potential Anti-HCV Agents
Source: Molecules. 2018 Nov 2;23(11):2863. doi: 10.3390/molecules23112863 (PMC6278568; doi:10.3390/molecules23112863)

iso-tyramine

Pulse Sequence: s2pul

UNITYplus-400 "unityplus400"
Date: Jan 17 2008
Solvent: DMSO
Ambient temperature
Total 64 repetitions

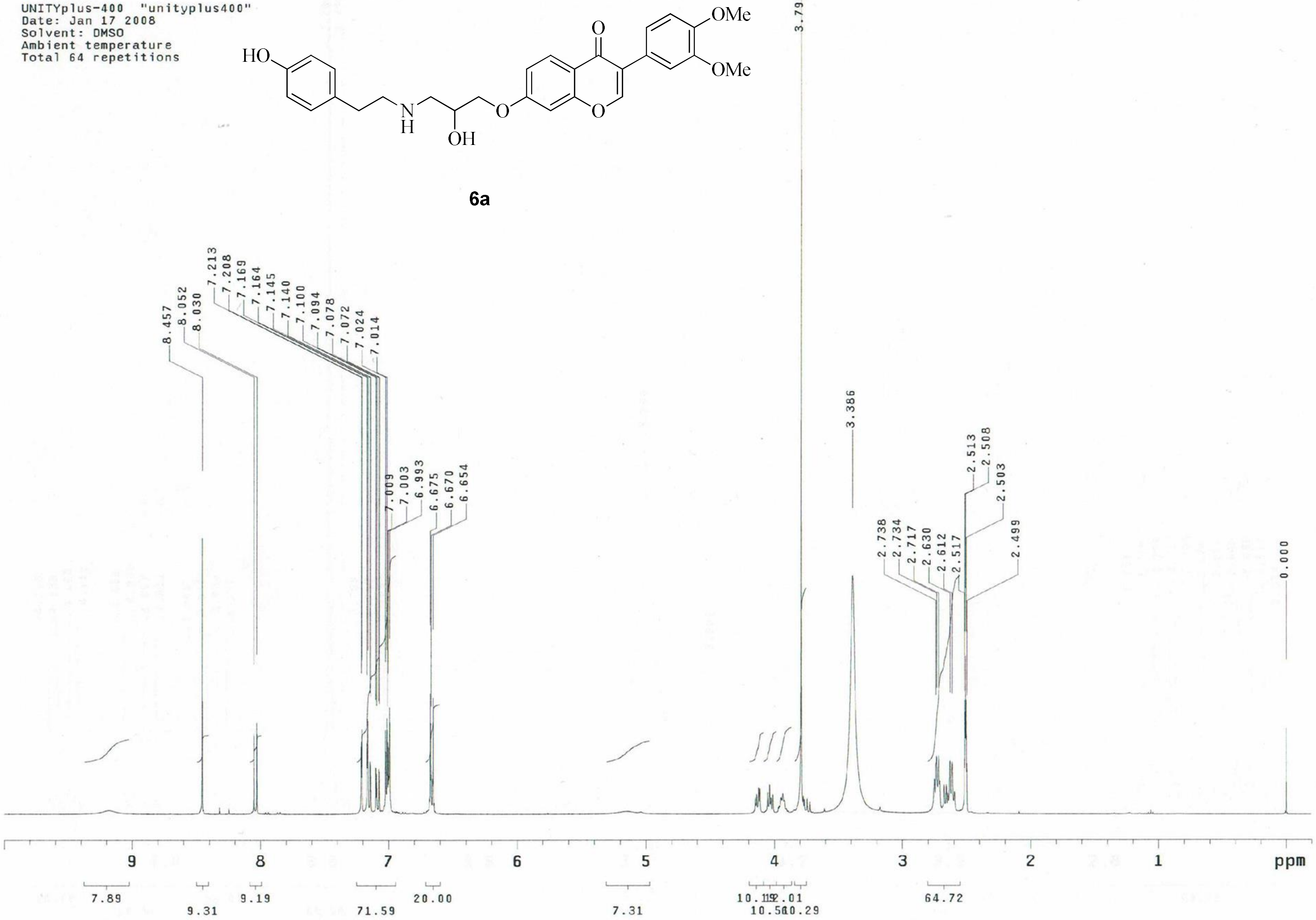

iso-tyramine

Pulse Sequence: s2pul
UNITYplus-400 "unityplus400"
Date: Jan 17 2008
Solvent: DMSO
Ambient temperature
Total 1664 repetitions

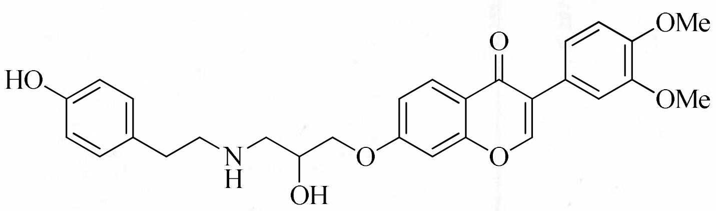

6a

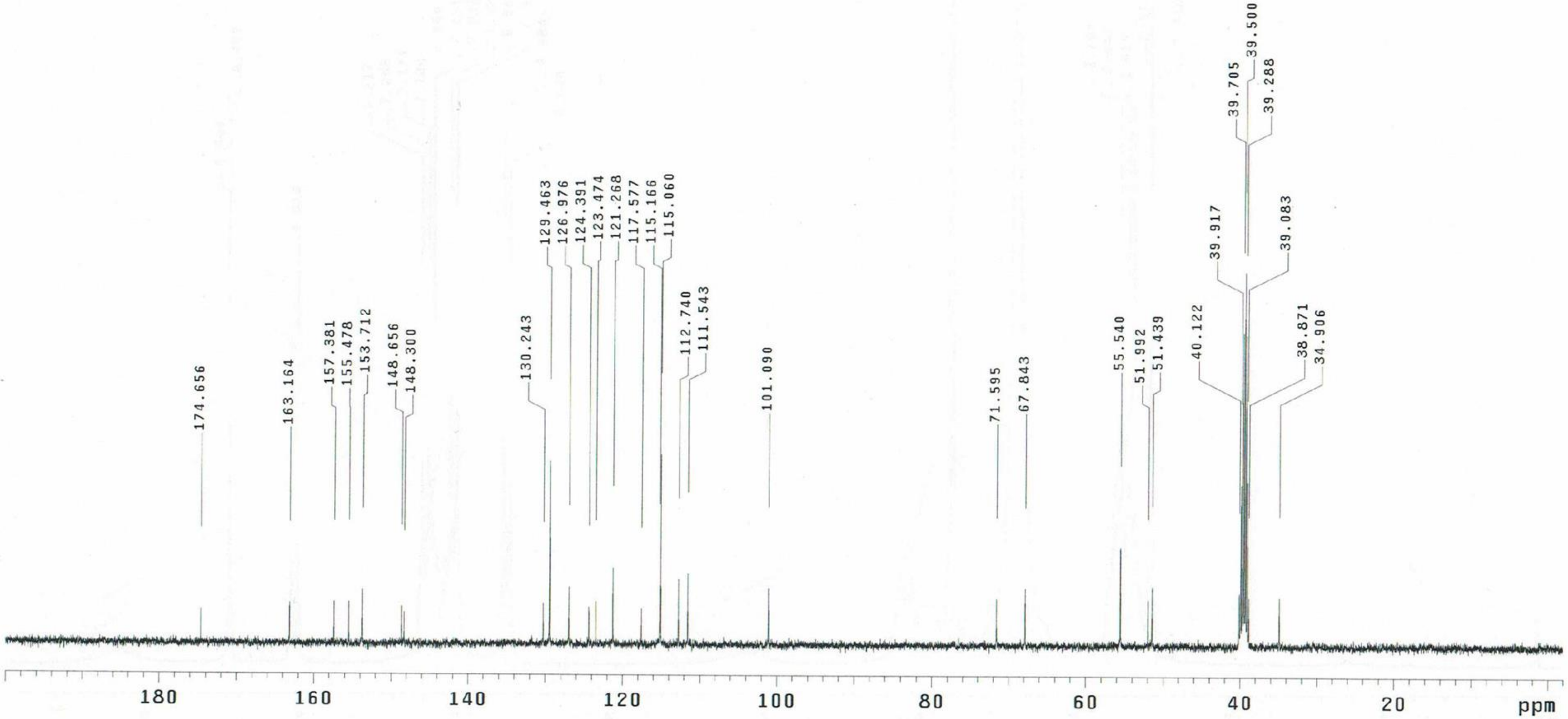

Molecules 2018, 23, 2863; doi:10.3390/molecules23112863

TCH-6b

Pulse Sequence: s2pul
Mercury-400BB "MerPlus400"
Date: Oct 11 2018
Solvent: cdcl3
Ambient temperature
Total 32 repetitions

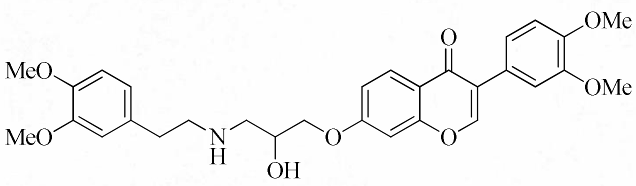

6b

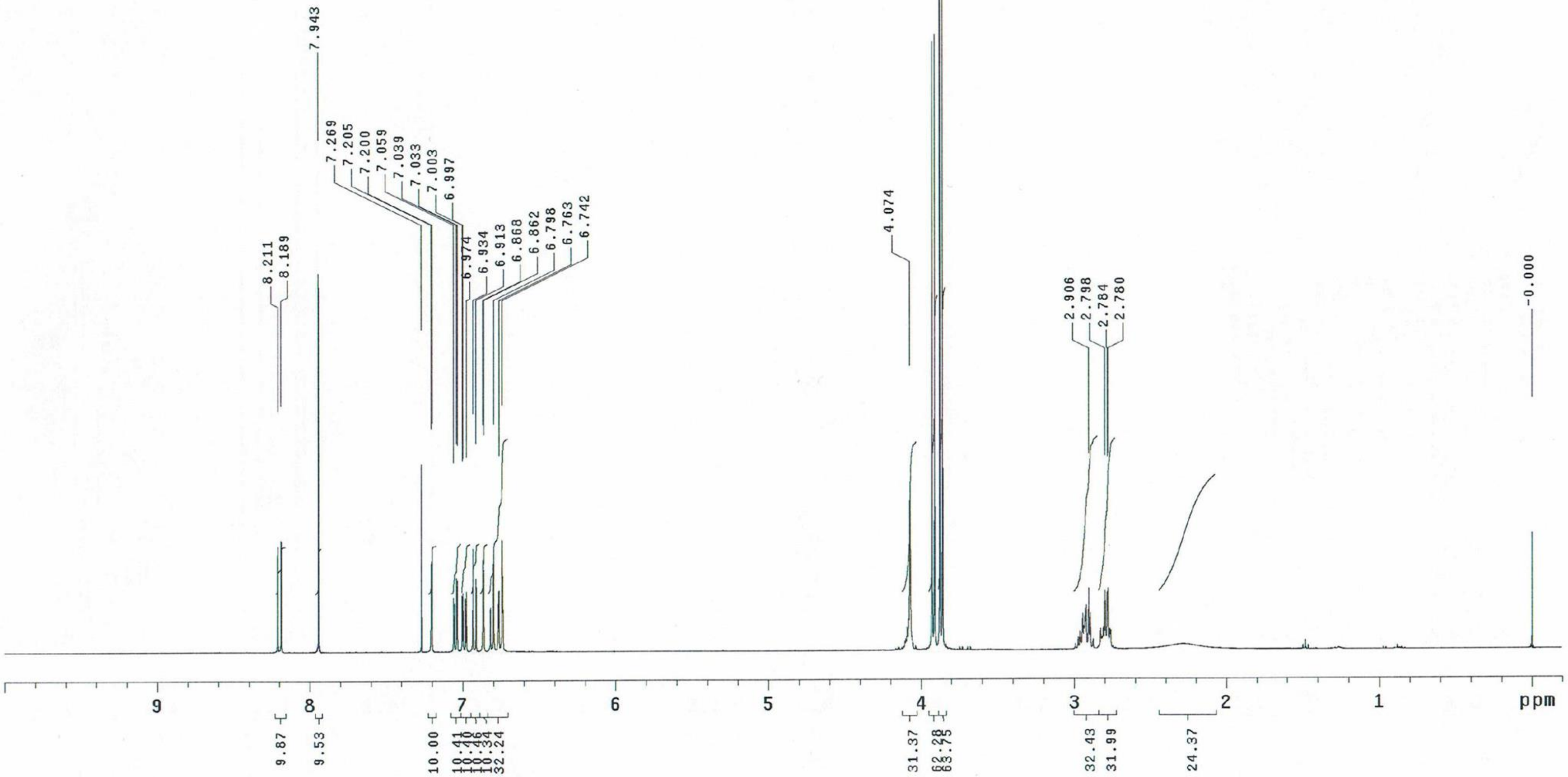

Molecules 2018, 23, 2863; doi:10.3390/molecules23112863

TCH-6b

Pulse Sequence: s2pul
Mercury-400BB "MerPlus400"
Date: Oct 11 2018
Solvent: cdcl3
Ambient temperature
Total 656 repetitions

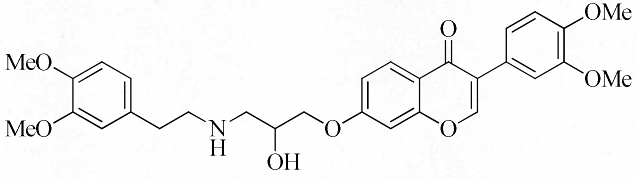

6b

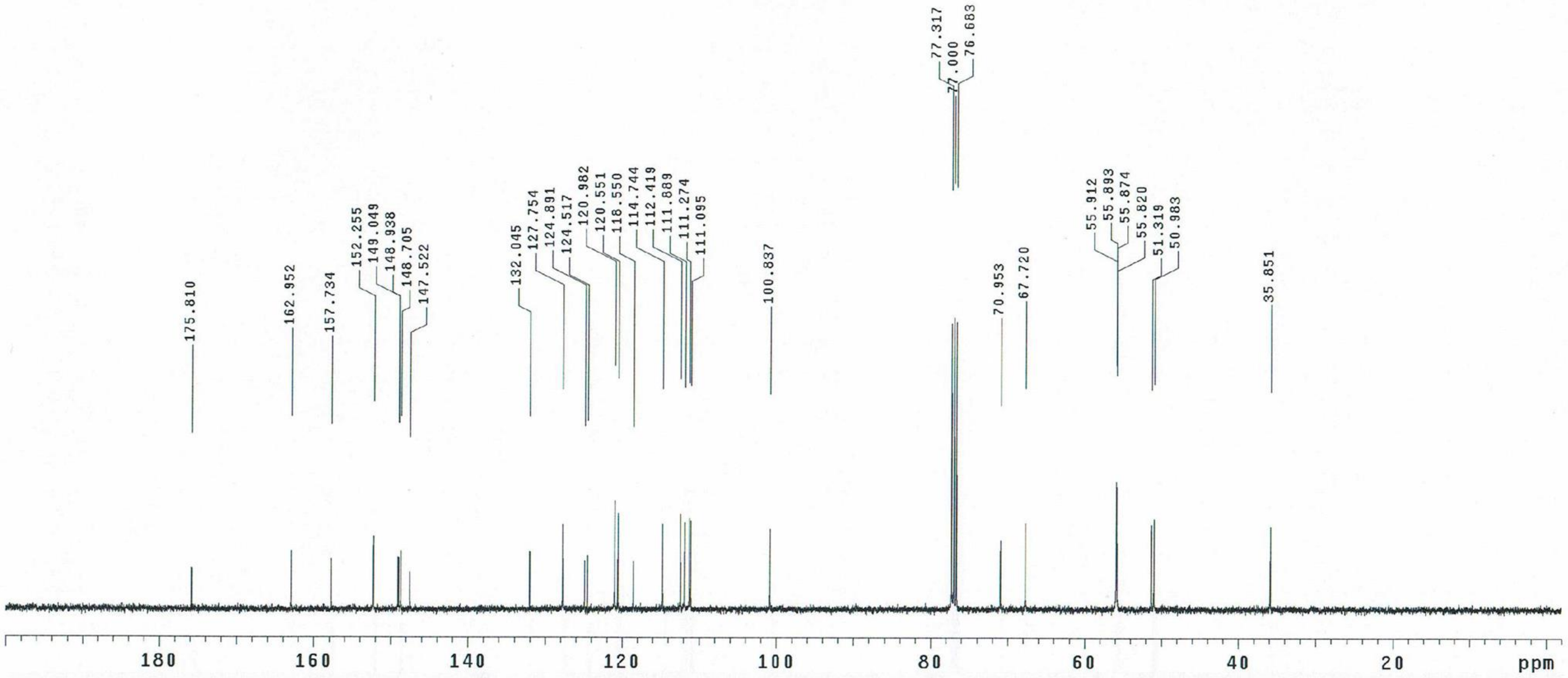

Molecules 2018, 23, 2863; doi:10.3390/molecules23112863

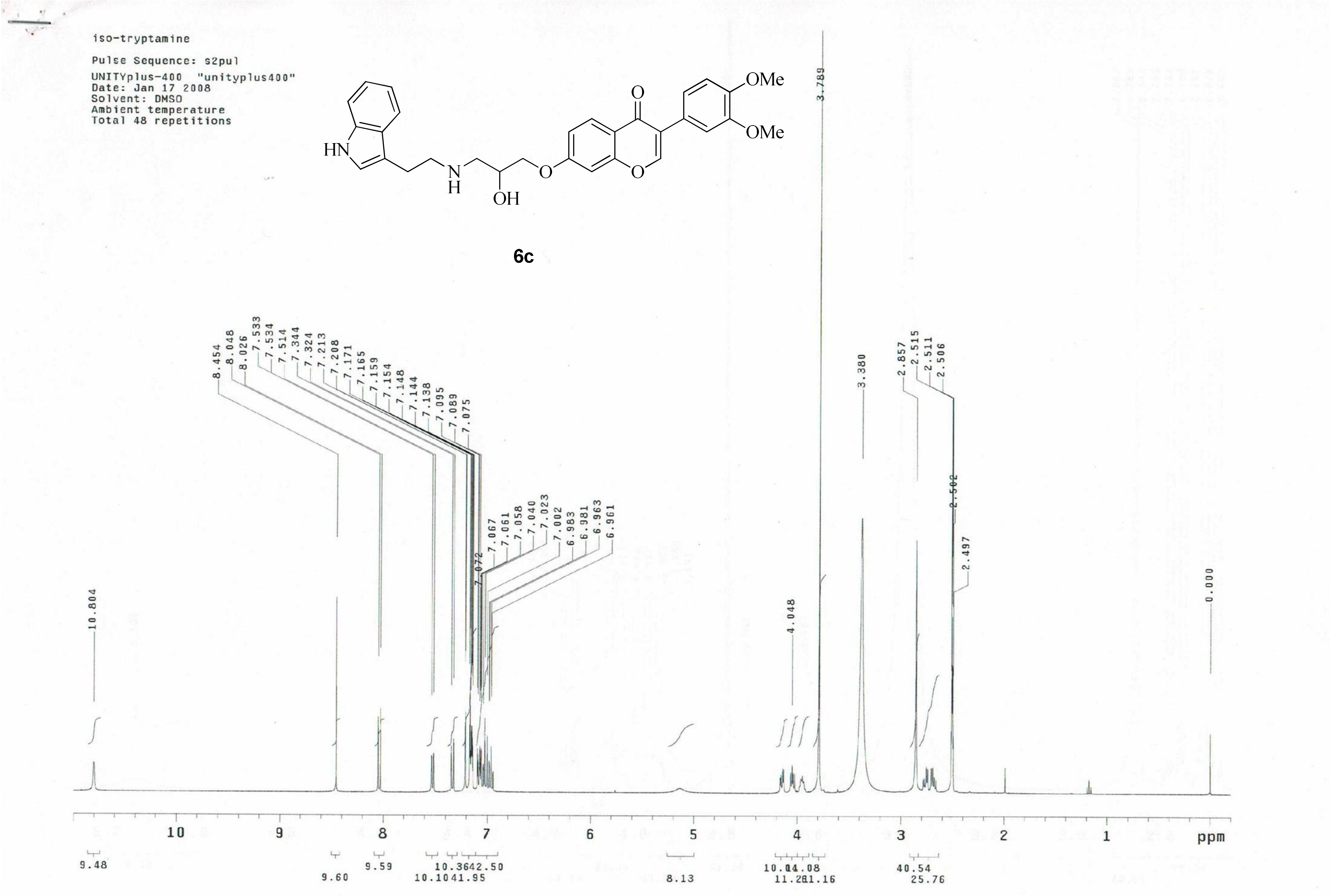

Molecules 2018, 23, 2863; doi:10.3390/molecules23112863

iso-tryptamine

Pulse Sequence: s2pul

UNITYplus-400 "unityplus400"
Date: Jan 17 2008
Solvent: DMSO
Ambient temperature
Total 5072 repetitions

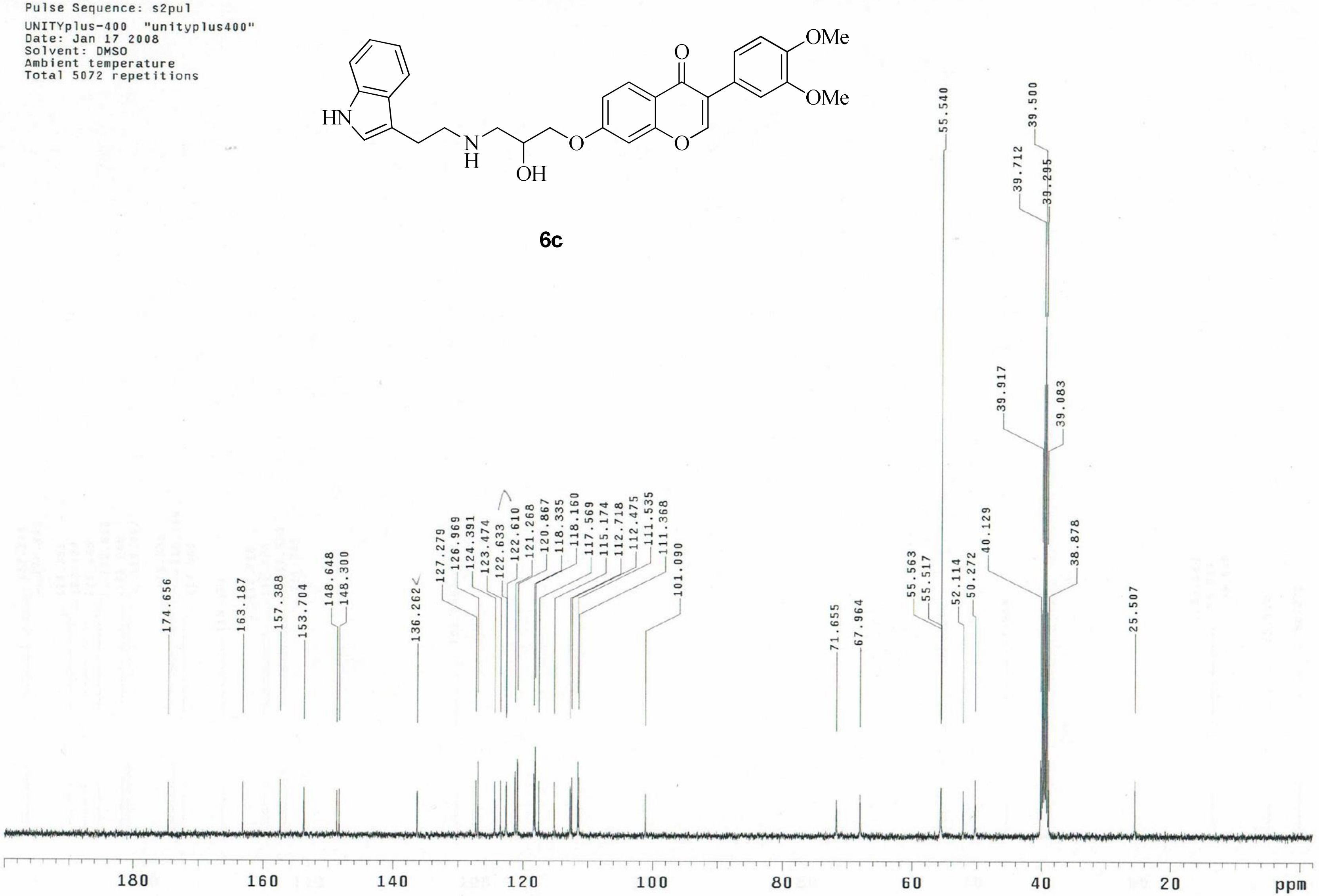

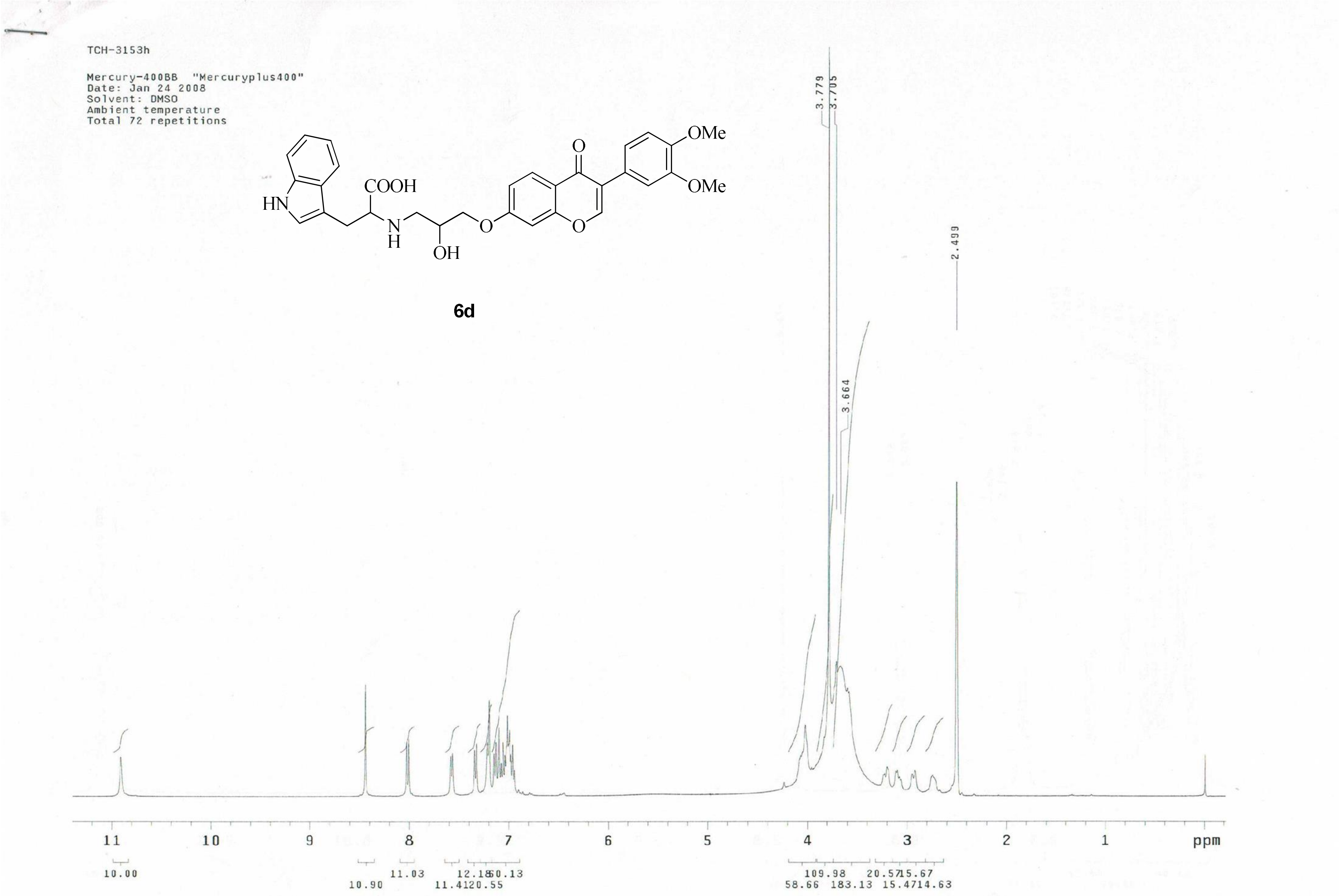
TCH-3153h
Mercury-400BB "Mercuryplus400"
Date: Jan 24 2008
Solvent: DMSO
Ambient temperature
Total 72 repetitions
COOH
HN
N
H
OH
O
OMe
OMe
6d
3.779
3.705
3.664
2.499
11
10
9
8
7
6
5
4
3
2
1
ppm
10.00
10.90
11.03
11.41
12.18
20.55
50.13
58.66
109.98
183.13
20.57
15.47
15.67
14.63

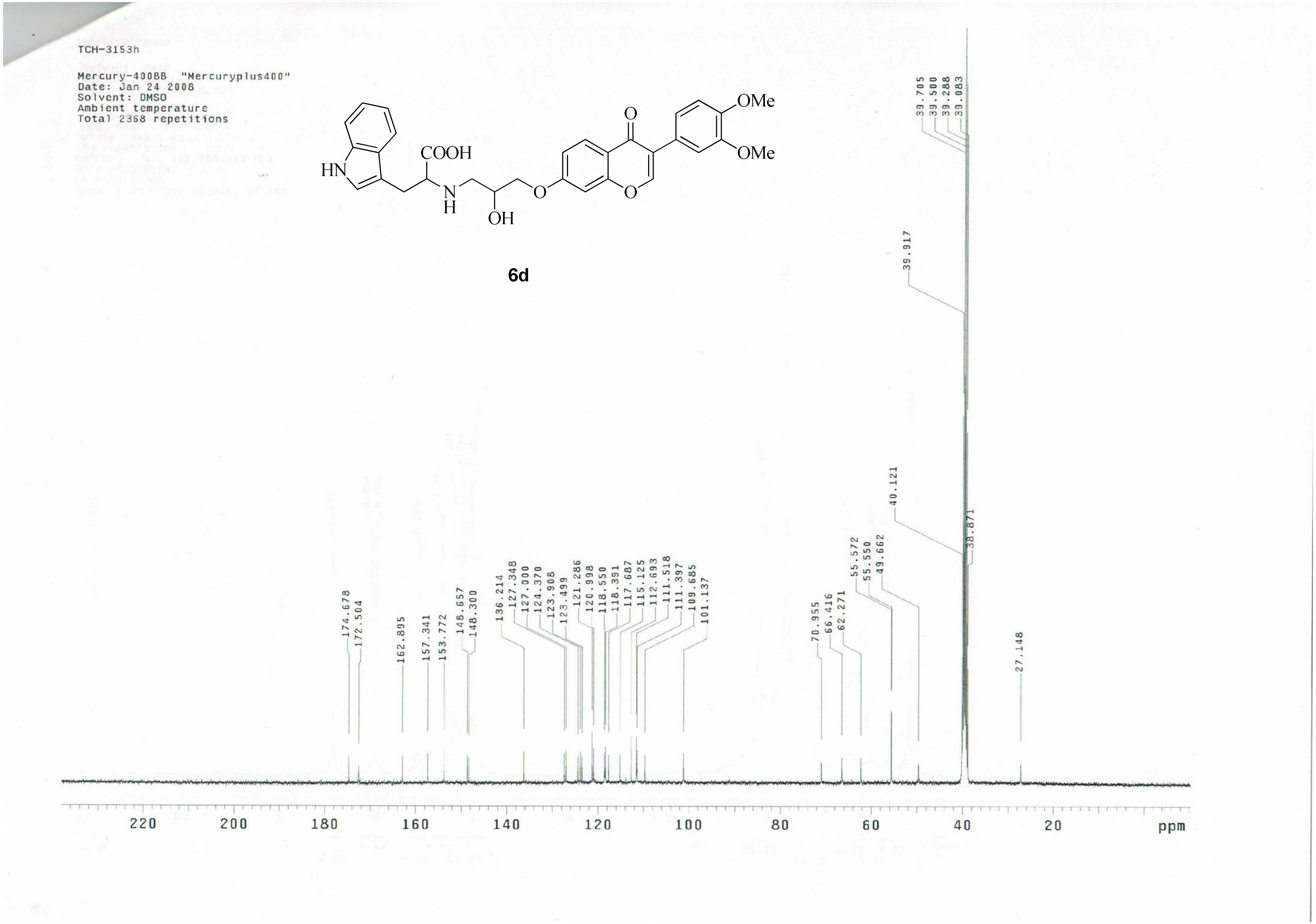

Molecules 2018, 23, 2863; doi:10.3390/molecules23112863

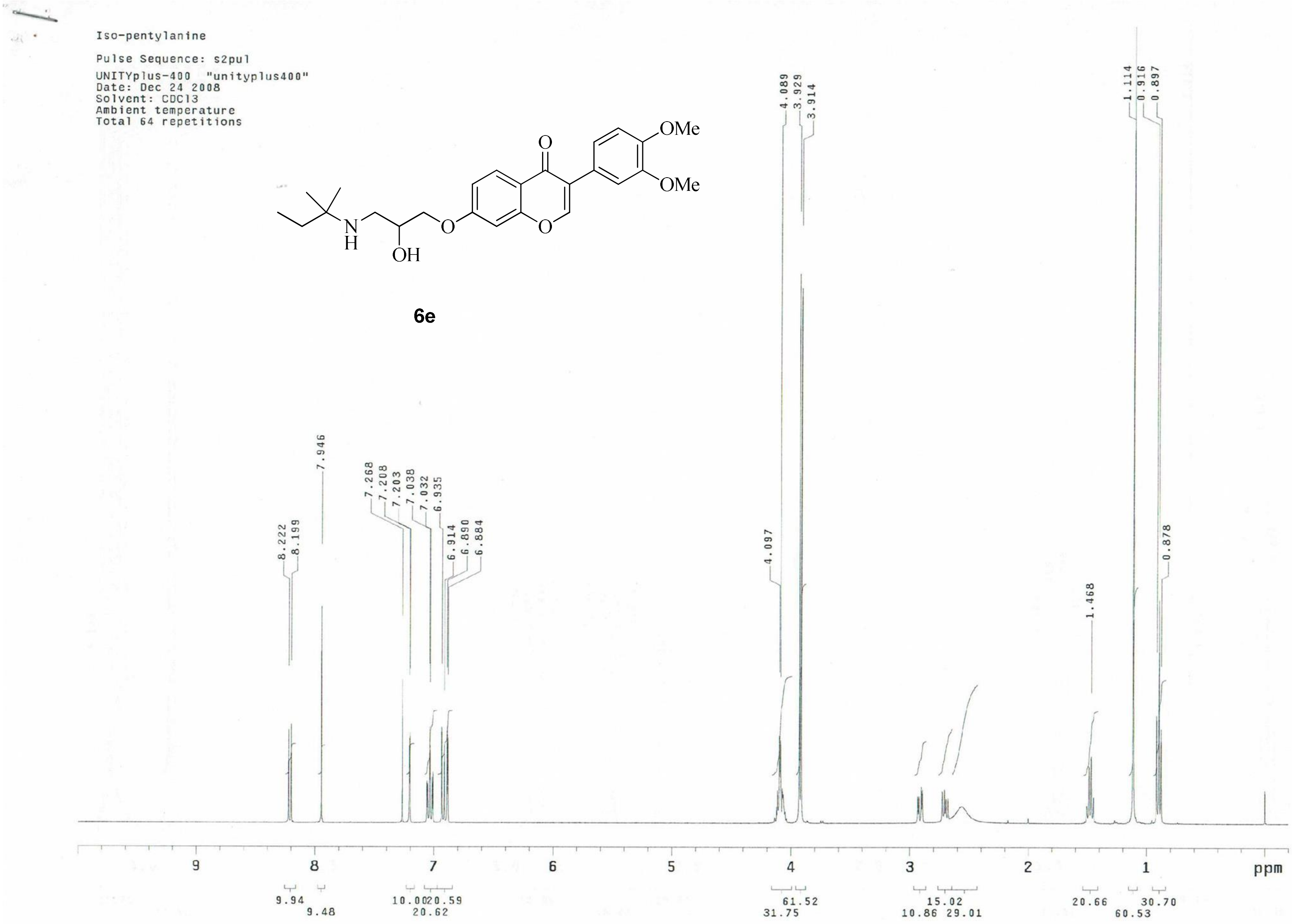
Iso-pentylanine
Pulse Sequence: s2pul
UNITYplus-400 "unityplus400"
Date: Dec 24 2008
Solvent: CDCl3
Ambient temperature
Total 64 repetitions
OMe
OMe
O
N
H
OH
O
O
6e
8.222
8.199
7.946
7.268
7.208
7.203
7.038
7.032
6.935
6.914
6.890
6.884
4.097
4.089
3.929
3.914
1.468
1.114
0.916
0.897
0.878
9
8
7
6
5
4
3
2
1
ppm
9.94
9.48
10.00
20.59
20.62
61.52
31.75
15.02
10.86
29.01
20.66
60.53
30.70

Iso-pentylanine

Pulse Sequence: s2pul

UNITYplus-400 "unityplus400"
Date: Dec 24 2008
Solvent: CDCl3
Ambient temperature
Total 2128 repetitions

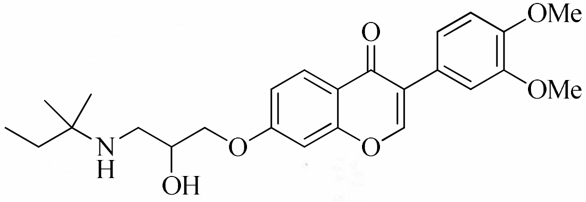

6e

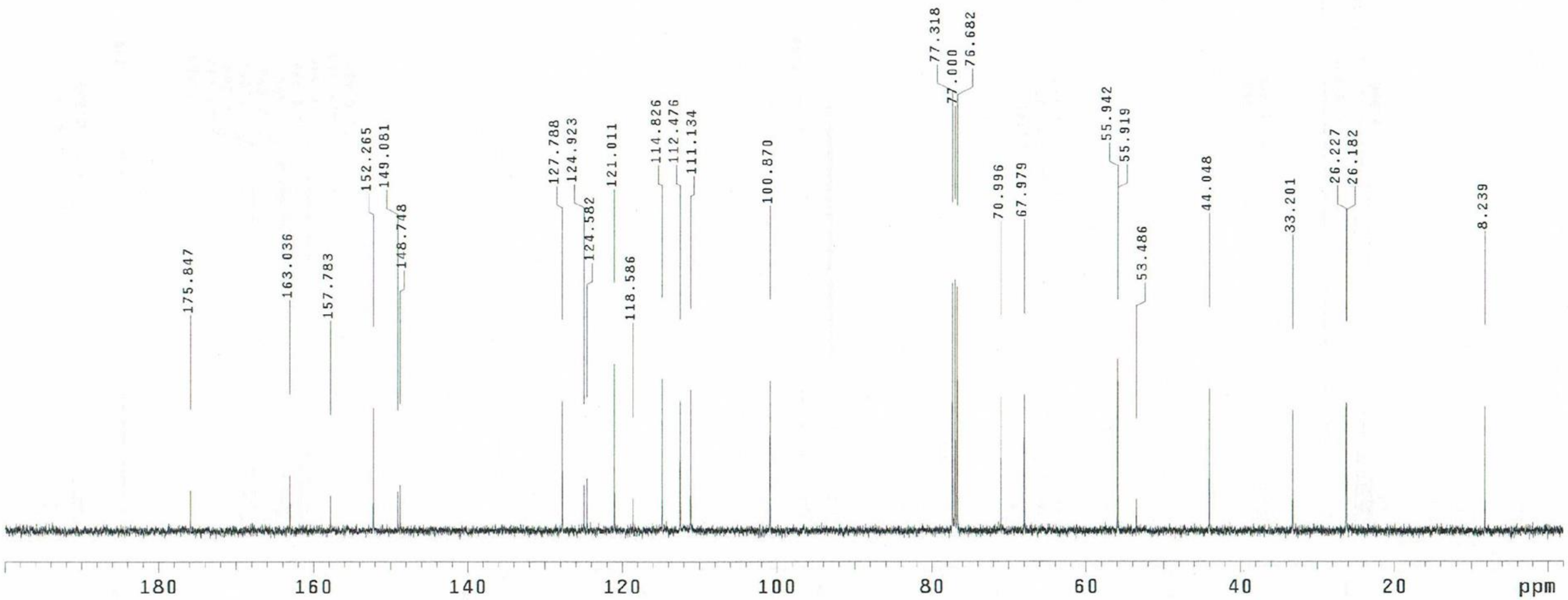

Molecules 2018, 23, 2863; doi:10.3390/molecules23112863

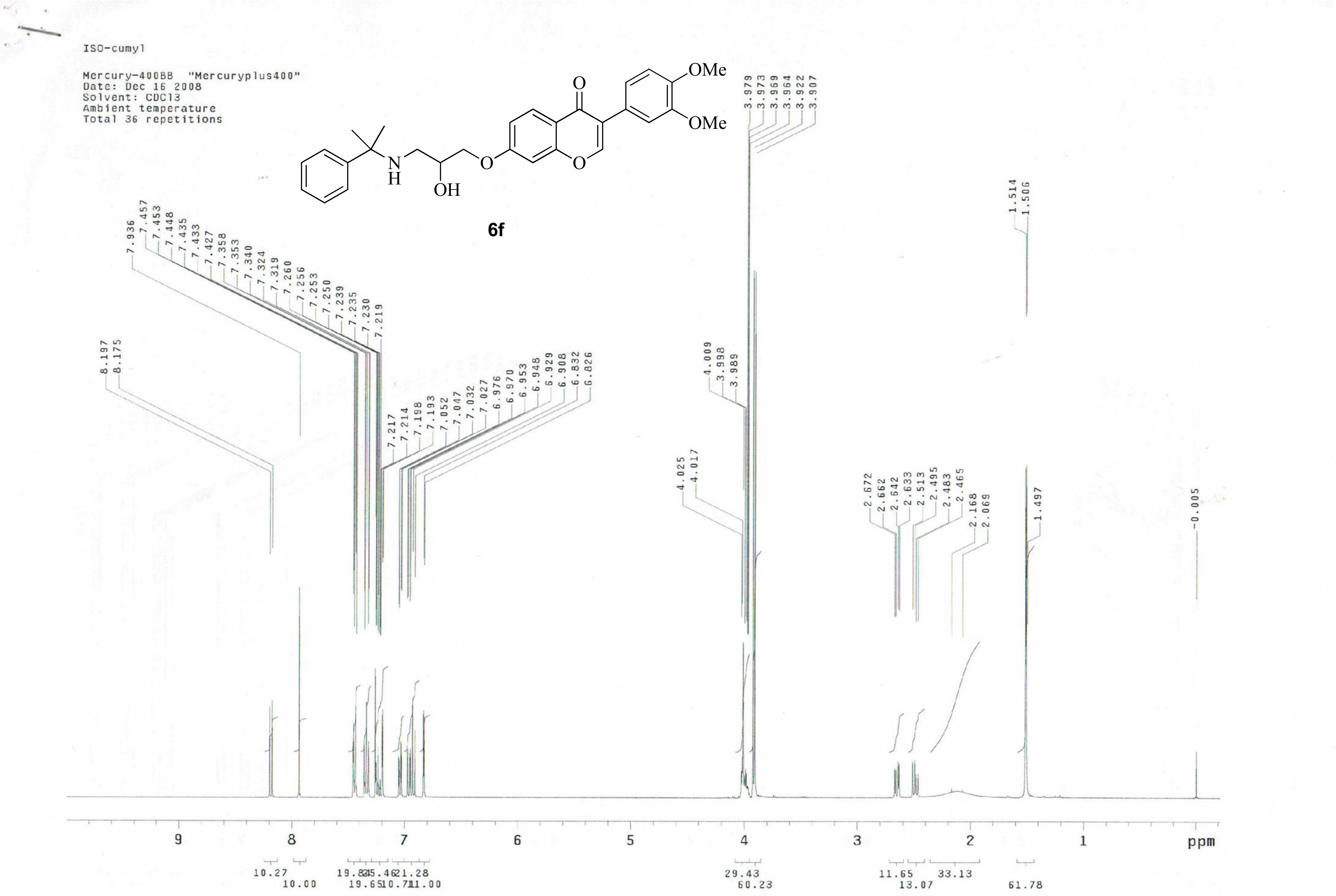
ISO-cumyl
Mercury-400BB "Mercuryplus400"
Date: Dec 16 2008
Solvent: CDCl3
Ambient temperature
Total 36 repetitions
OMe
OMe
O
O
O
N
H
OH
6f
8.197
8.175
7.936
7.457
7.453
7.448
7.435
7.433
7.427
7.358
7.353
7.340
7.324
7.319
7.260
7.256
7.253
7.250
7.239
7.235
7.230
7.219
7.217
7.214
7.198
7.193
7.052
7.047
7.032
7.027
6.976
6.970
6.953
6.948
6.929
6.908
6.832
6.826
4.025
4.017
4.009
3.998
3.989
3.979
3.973
3.969
3.964
3.922
3.907
2.672
2.662
2.642
2.633
2.513
2.495
2.483
2.465
2.168
2.069
1.514
1.506
1.497
-0.005
9
8
7
6
5
4
3
2
1
ppm
10.27
10.00
19.65
10.71
11.00
21.28
29.43
60.23
11.65
13.07
33.13
61.78

ISO-cumyl

Mercury-400BB "Mercuryplus400"
Date: Dec 16 2008
Solvent: CDCl3
Ambient temperature
Total 1072 repetitions

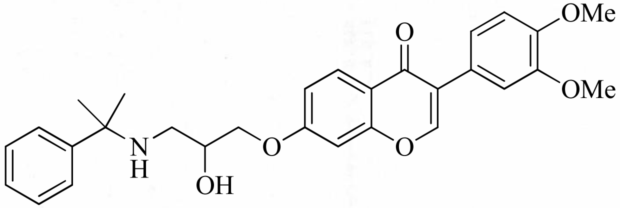

6f

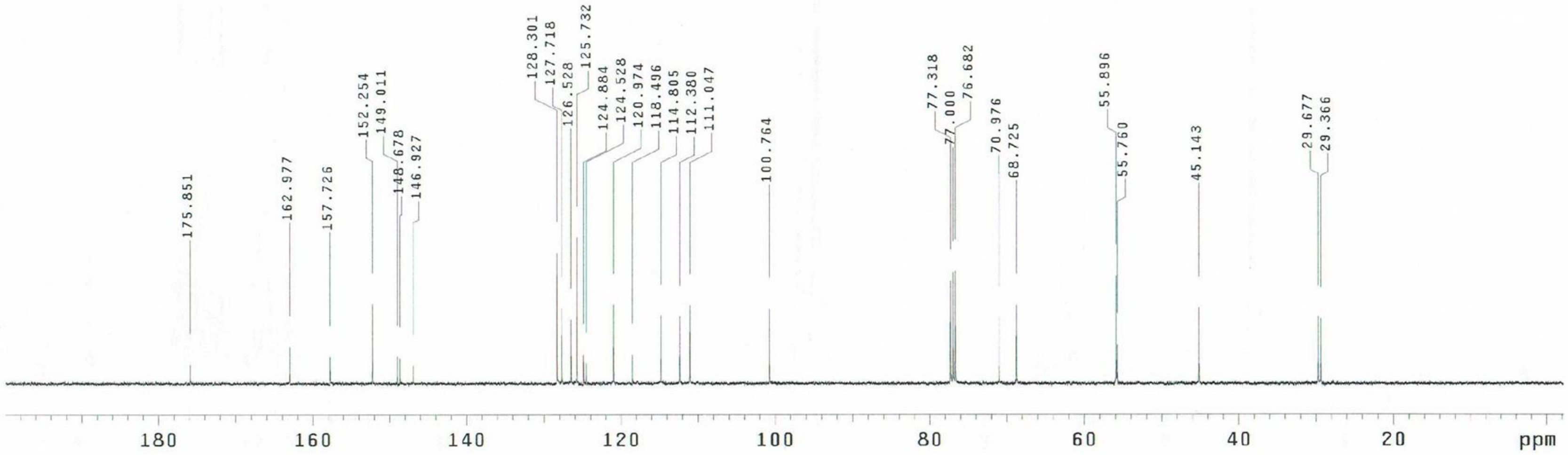

Molecules 2018, 23, 2863; doi:10.3390/molecules23112863

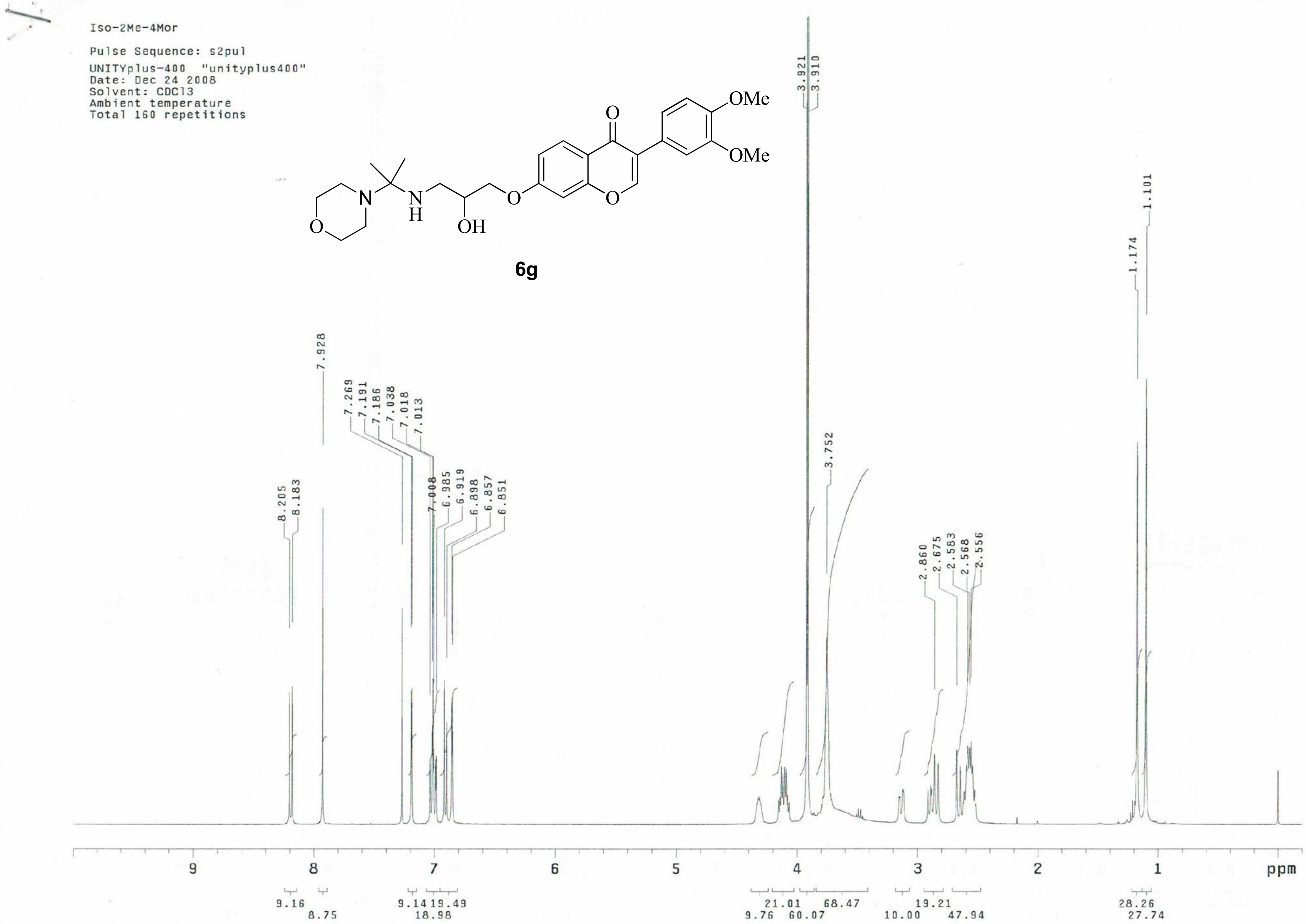

Molecules 2018, 23, 2863; doi:10.3390/molecules23112863

Iso-2Me-4Mor

Pulse Sequence: s2pul

UNITYplus-400 "unityplus400"
Date: Dec 24 2008
Solvent: CDCl3
Ambient temperature
Total 1840 repetitions

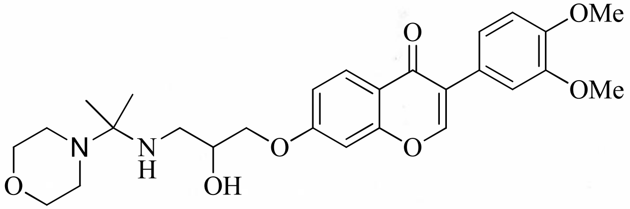

6g

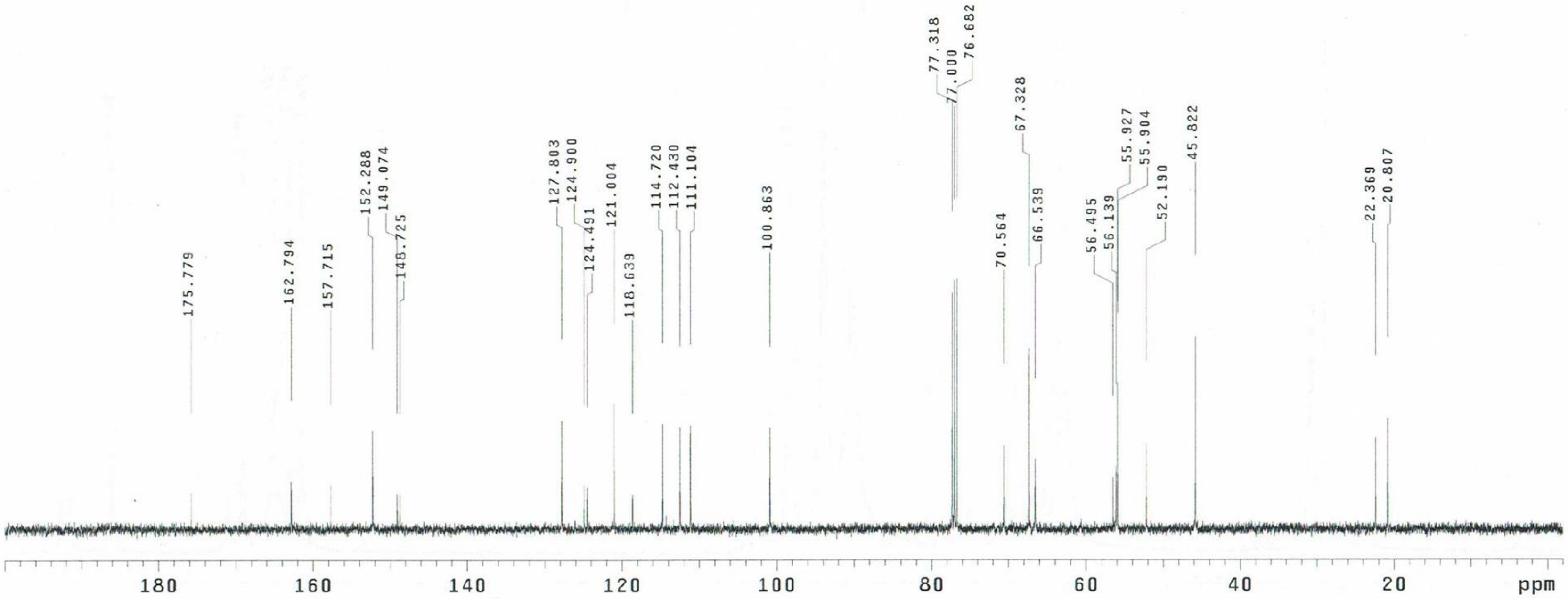

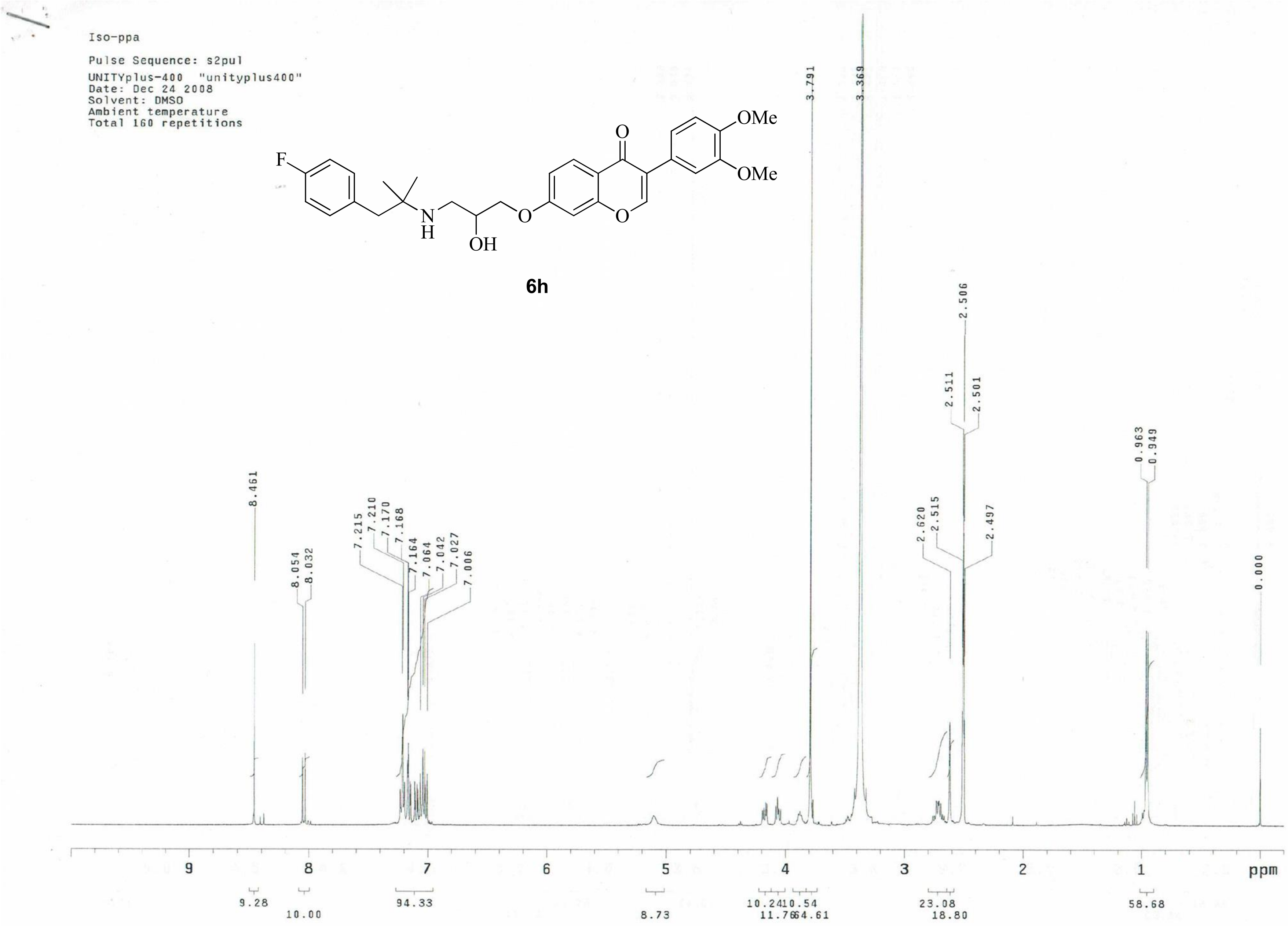
Iso-ppa
Pulse Sequence: s2pul
UNITYplus-400 "unityplus400"
Date: Dec 24 2008
Solvent: DMSO
Ambient temperature
Total 160 repetitions
6h
ppm

Iso-ppa
Pulse Sequence: s2pul
UNITYplus-400 "unityplus400"
Date: Dec 24 2008
Solvent: DMSO
Ambient temperature
Total 64000 repetitions

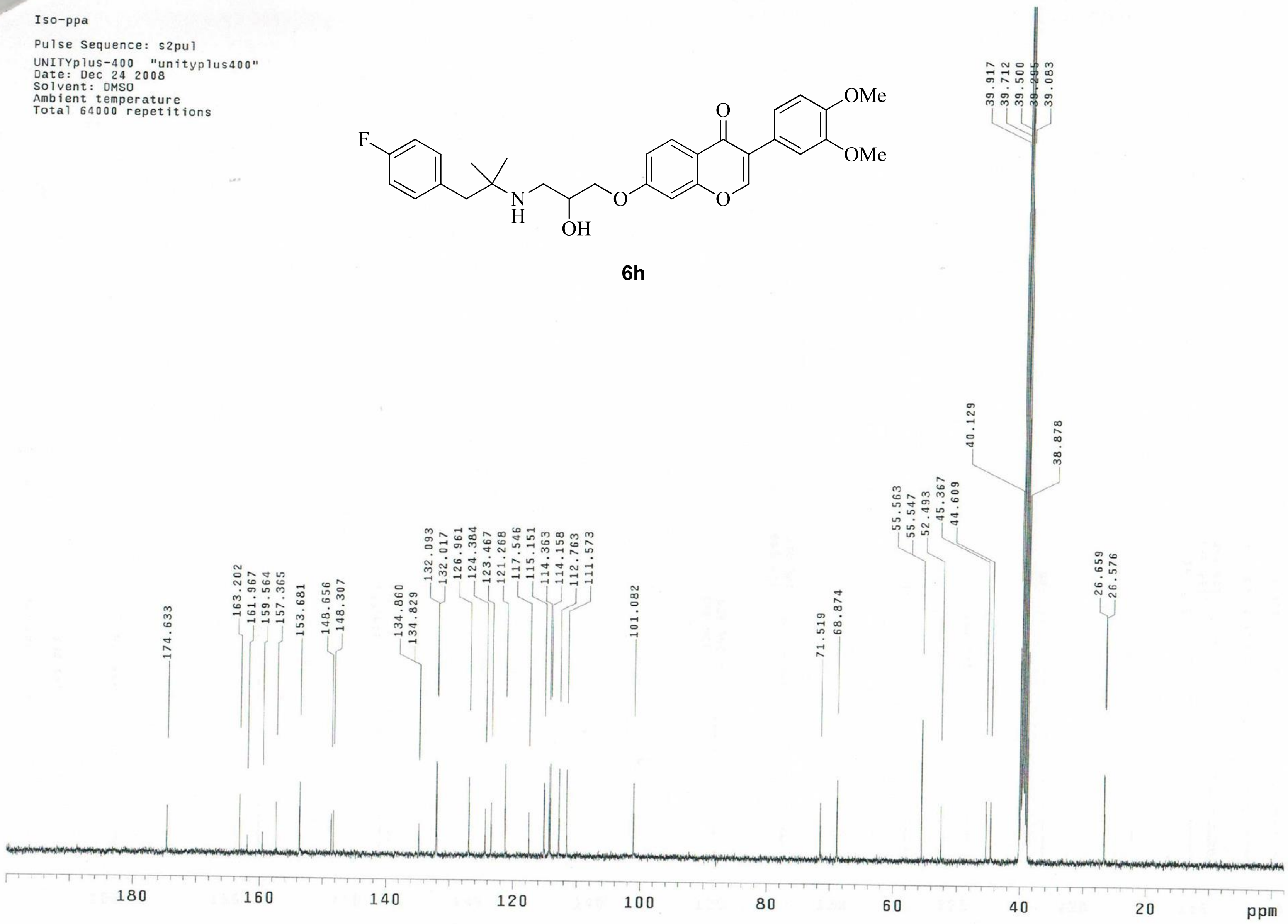

Molecules 2018, 23, 2863; doi:10.3390/molecules23112863

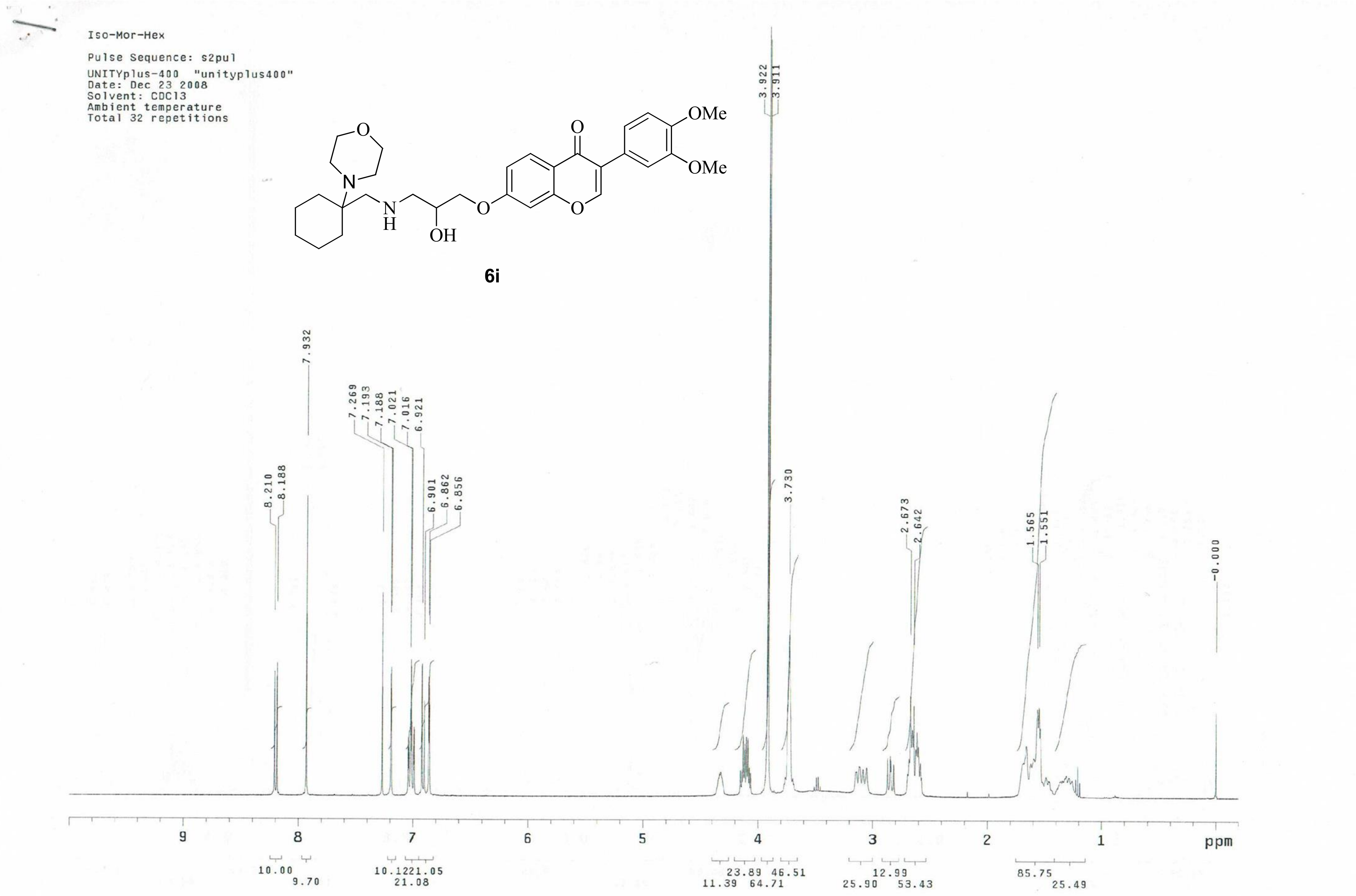
Iso-Mor-Hex
Pulse Sequence: s2pul
UNITYplus-400 "unityplus400"
Date: Dec 23 2008
Solvent: CDCl3
Ambient temperature
Total 32 repetitions
O
OMe
OMe
N
O
N
H
OH
O
O
6i
8.210
8.188
7.932
7.269
7.193
7.188
7.021
7.016
6.921
6.901
6.862
6.856
3.922
3.911
3.730
2.673
2.642
1.565
1.551
-0.000
9
8
7
6
5
4
3
2
1
ppm
10.00
9.70
10.12
21.05
21.08
23.89
46.51
11.39
64.71
12.99
25.90
53.43
85.75
25.49

Iso-Mor-Hex
Pulse Sequence: s2pul
UNITYplus-400 "unityplus400"
Date: Dec 23 2008
Solvent: CDCl3
Ambient temperature
Total 2848 repetitions

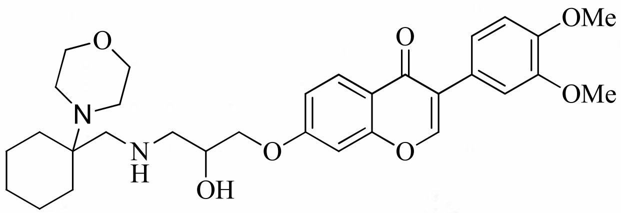

6i

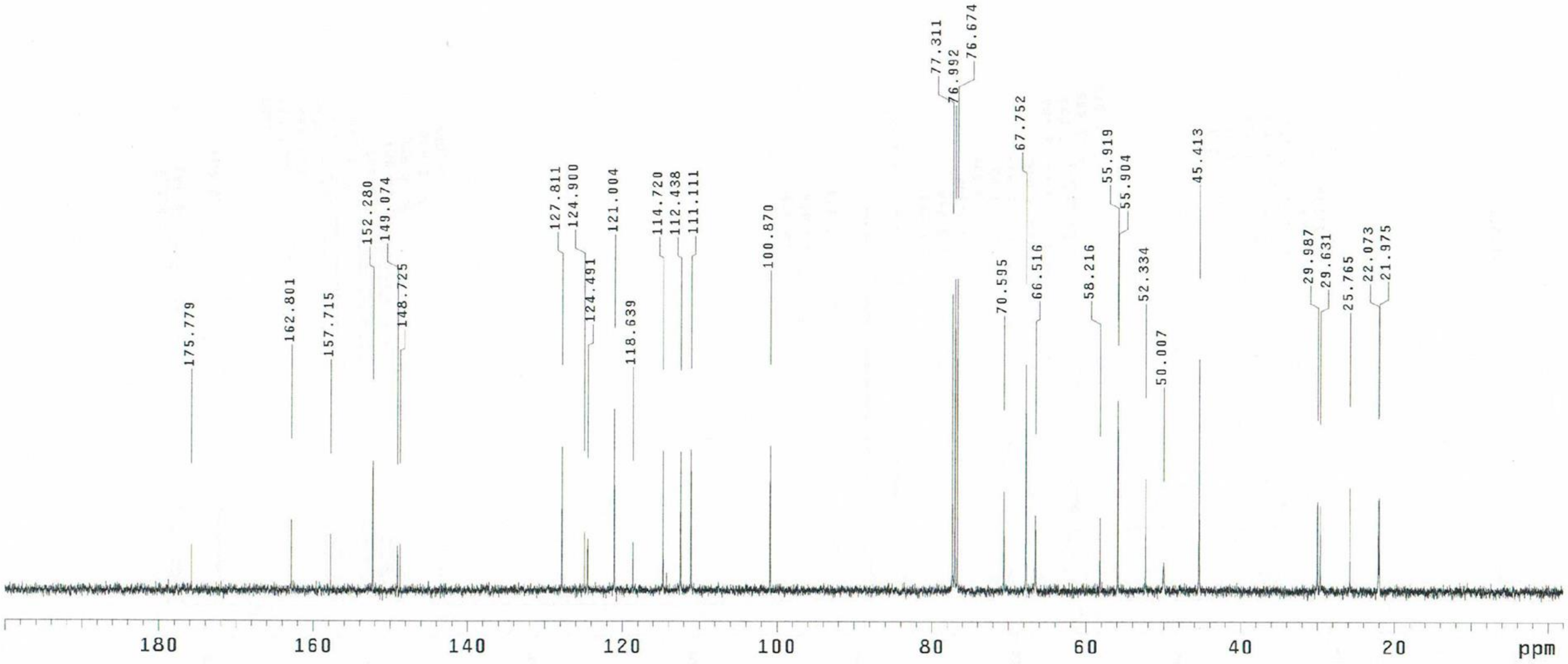

Supplement: Supplementary file 1 [file molecules-23-02863-s001.pdf]
